# Massive infection of a song thrush by *Mesocestoides* sp. (Cestoda) tetrathyridia that genetically match acephalic metacestodes causing lethal peritoneal larval cestodiasis in domesticated mammals

**DOI:** 10.1186/s13071-019-3480-1

**Published:** 2019-05-14

**Authors:** Petr Heneberg, Boyko B. Georgiev, Jiljí Sitko, Ivan Literák

**Affiliations:** 1 Third Faculty of Medicine, Charles University, Prague, Czechia; 2 Institute of Biodiversity and Ecosystem Research, Bulgarian Academy of Sciences, Sofia, Bulgaria; 3 Comenius Museum, Moravian Ornithological Station, Přerov, Czechia; 4 Department of Biology and Wildlife Diseases, Faculty of Veterinary Hygiene and Ecology, University of Veterinary and Pharmaceutical Sciences Brno, Brno, Czechia

**Keywords:** Acephalic metacestodes, Cestoda, Lethal infection, *Mesocestoides*, Phylogenetic analysis, Tetrathyridia

## Abstract

**Background:**

Peritoneal larval cestodiasis induced by *Mesocestoides* Vaillant, 1863 (Cyclophyllidea: Mesocestoididae) is a common cause of severe infections in domestic dogs and cats, reported also from other mammals and less frequently from birds. However, there is a limited knowledge on the taxonomy of causative agents of this disease.

**Results:**

In the present study, we investigated a massive, likely lethal, infection of a song thrush *Turdus philomelos* (Passeriformes: Turdidae) by *Mesocestoides* sp. tetrathyridia. We performed combined morphological and phylogenetic analysis of the tetrathyridia and compared them with the materials obtained previously from other birds and mammals. The metrical data fitted within the wide range reported by previous authors but confirmed the limited value of morphological data for species identification of tetrathyridia of *Mesocestoides* spp. The molecular analyses suggested that the isolates represented an unidentified *Mesocestoides* sp. that was previously repeatedly isolated and sequenced in larval and adult forms from domestic dogs and cats in Europe, the Middle East and North Africa. In contrast to the present study, which found encysted tetrathyridia, four of the five previous studies that identified the same species described infections by acephalic metacestodes only.

**Conclusions:**

The tetrathyridia of the examined *Mesocestoides* sp. are described in the present study for the first time. However, the possible match with the species that were previously reported to infect birds remains uncertain. The phylogenetic analyses also suggested the rejection of two cases that were previously identified as *Mesocestoides corti* as they were likely caused by the same species as in the presently reported infection case. The newly provided DNA sequences should allow the assignment to species in the future, when adults of the genus *Mesocestoides* are more thoroughly sequenced.

**Electronic supplementary material:**

The online version of this article (10.1186/s13071-019-3480-1) contains supplementary material, which is available to authorized users.

## Background

Adult cestodes of the genus *Mesocestoides* Vaillant, 1863 (Cyclophyllidea: Mesocestoididae) are intestinal parasites of carnivore mammals (canids, felids, mustelids or hyaenids) and rarely birds of prey (e.g. [1–4]). *Mesocestoides* spp. have zoonotic potential; records of the infections of non-human primates are frequent [5], but records of infections of humans are sporadic [6, 7]. In addition to the adult individuals, the juvenile stages, commonly known as tetrathyridia and acephalic metacestodes, commonly parasitize a broad range of mammals, and sometimes reptiles, amphibians and birds. The first intermediate host of *Mesocestoides* spp. is unknown, likely invertebrates [8, 9]. However, McAllister et al. [10] recently suggested that the hypothetical first invertebrate host may be absent and *Mesocestoides* spp. may develop through a two-host life-cycle only, using vertebrates as the intermediate host. *Mesocestoides* spp. have been recognized for their problematic taxonomic status and the lack of good morphological diagnostic features, with the validity of many species being questioned by numerous authors until the onset of genetic evaluations [11, 12]. In birds, the records of *Mesocestoides* spp. larvae are rare and are not limited to any family or order [8, 13–18]. However, occasionally the prevalence can be massive, such as in the *Alectoris graeca* populations examined in Greece [19]. The infections in birds may affect various tissues and organs. The body cavity is the most common infection site, where *Mesocestoides* larvae may cause disease termed peritoneal larval cestodiasis [13, 17, 20–23]. However, other sites may also become infected; these include the peritoneum [20], lungs [20], air sacs [13], intercostal muscles [15], liver [20, 22] and subcutaneous connective tissue [22]. The tetrathyridia could also be present in cutaneous cysts [17].

With regards to studies that employed DNA sequencing, several DNA loci were examined, including *12S* ribosomal deoxyribonucleic acid (rDNA) [10, 12, 24–30], cytochrome *c* oxidase subunit 1 (*cox*1) [10, 24, 25, 28, 30–34], nicotinamide adenine dinucleotide dehydrogenase subunit 1 (*nad*1) [10, 28, 32, 34], *18S* rDNA [29, 32, 35–37], internal transcribed spacer 2 (ITS2) [12, 35] and *28S* rDNA [24, 32, 37, 38]. Sequences of *Mesocestoides* spp. from bird hosts were provided by Literák et al. [17], who sequenced *18S* rDNA of *Mesocestoides* sp. from thoracic cavity of a common starling, *Sturnus vulgaris*, from Czechia, as well as by Skirnisson et al. [24] reporting several loci of tetrathyridia from the rock ptarmigan, *Lagopus muta*, from Iceland. Despite the voluminous literature, phylogenetic analyses indisputably recognize only *M. canislagopodis*, *M. litteratus*, *Mesocestoides lineatus* (Goeze, 1782) and *Mesocestoides corti* Hoeppli, 1925/*M*. *vogae* Etges, 1991 [24], whereas the number of morphospecies varies between 12 [15] and 27 [39]. Moreover, the names *M. corti* and *M. vogae* were used for larval stages only and their taxonomic status remains uncertain [16].

In the present study, we aimed to investigate a massive and lethal infection of a song thrush, *Turdus philomelos*, by *Mesocestoides* sp. tetrathyridia. We performed combined morphological and phylogenetic analysis of the tetrathyridia and compared them with the materials obtained previously from other birds and mammals, identifying a match with a large number of isolates that were recently reported from domestic dogs and cats across Europe, the Middle East and North Africa.

## Results

The examined *T. philomelos* had cysts of *Mesocestoides* sp. tetrathyridia across the whole body cavity, from the surface of lungs through liver, intestine and kidneys. The tetrathyridia were encysted individually or in small groups up to five individuals in a single cyst; in total, 104 tetrathyridia were present. The examined bird displayed signs of emaciation, the ovary was defunct and the eggs were up to 1 mm in diameter.

**Fig. 1:**
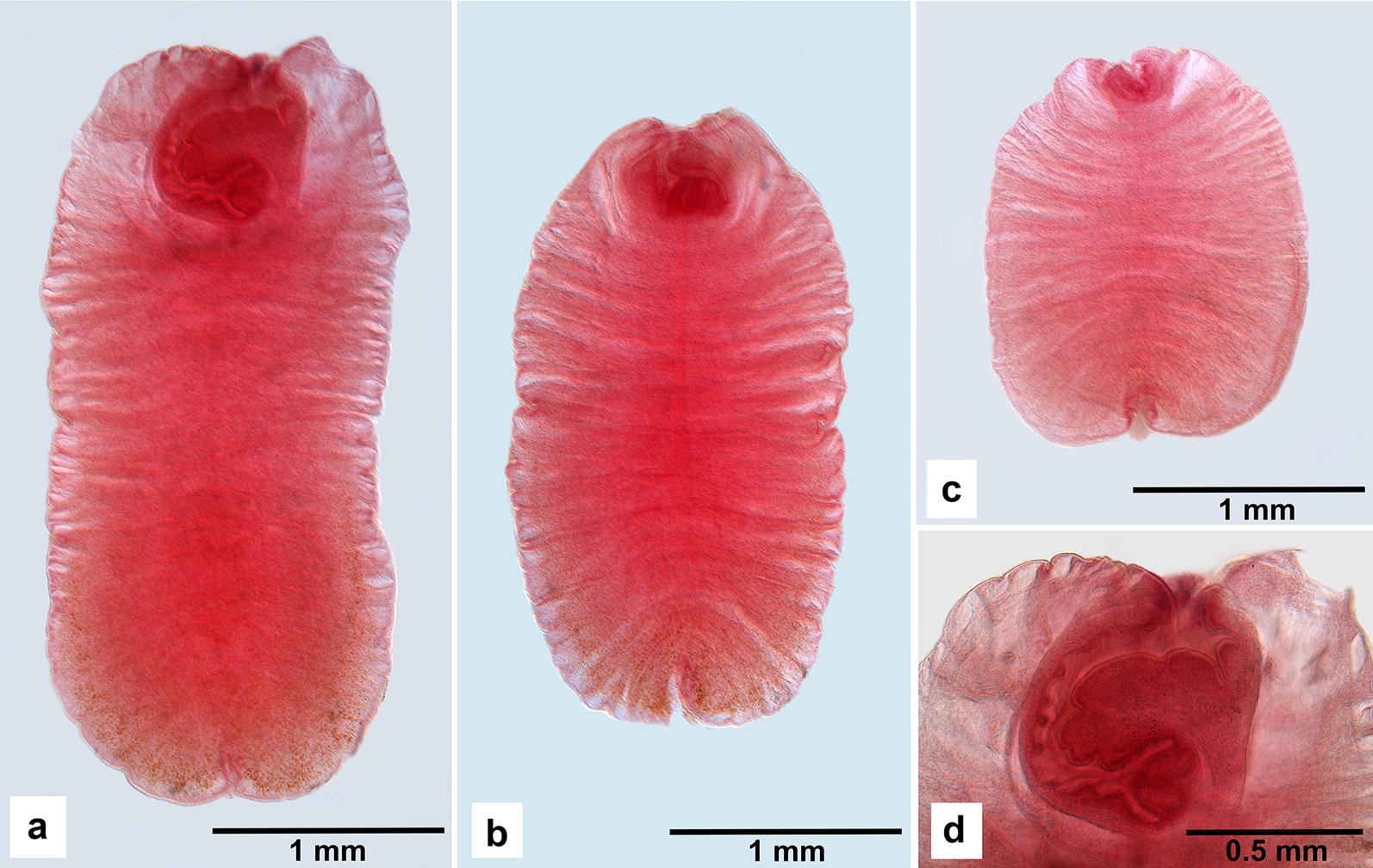
Tetrathyridia of *Mesocestoides* sp. found in the body cavity of *Turdus philomelos* in Czechia (iron acetocarmine, dimethyl phthalate, Canada balsam). **a**–**c** Individuals demonstrating variations in size and shape of tetrathyridia. Note the invaginated scolex and neck (transformed into “anterior canal”) as well as the presence of posterior invagination in all individuals. **d** Anterior end of the individual presented in **a**. Note the curved “anterior canal” representing the scolex and the neck invaginated into the body of the metacestode. The apical part of the scolex bearing suckers is distinct at the bottom of the canal

The morphologically examined tetrathyridia were 1641–4375 μm (2727 ± 597 μm, *n* = 36) long and 1247–1903 μm (1592 ± 122 μm, *n* = 36) wide. Their body was always longer than wide (Fig. 1a–c), with a length to width ratio of 1.03–2.67 μm (1.71 ± 0.31 μm, *n* = 36). Anterior end was invaginated. In 72% of individuals, scolex and neck were invaginated into body of metacestode, thus forming curved anterior canal (Fig. 1d) with variable length and width, 202–975 μm (546 ± 154 μm, *n* = 26) long and 36–138 μm (72 ± 21 μm, *n* = 26) wide, with suckers often forming its bottom. Suckers were oval, with diameter 121–201 μm (165 ± 14 μm, *n* = 34). Posterior end of body was invaginated, with pore of osmoregulatory system sometimes distinct in middle of invagination.

**Fig. 2:**
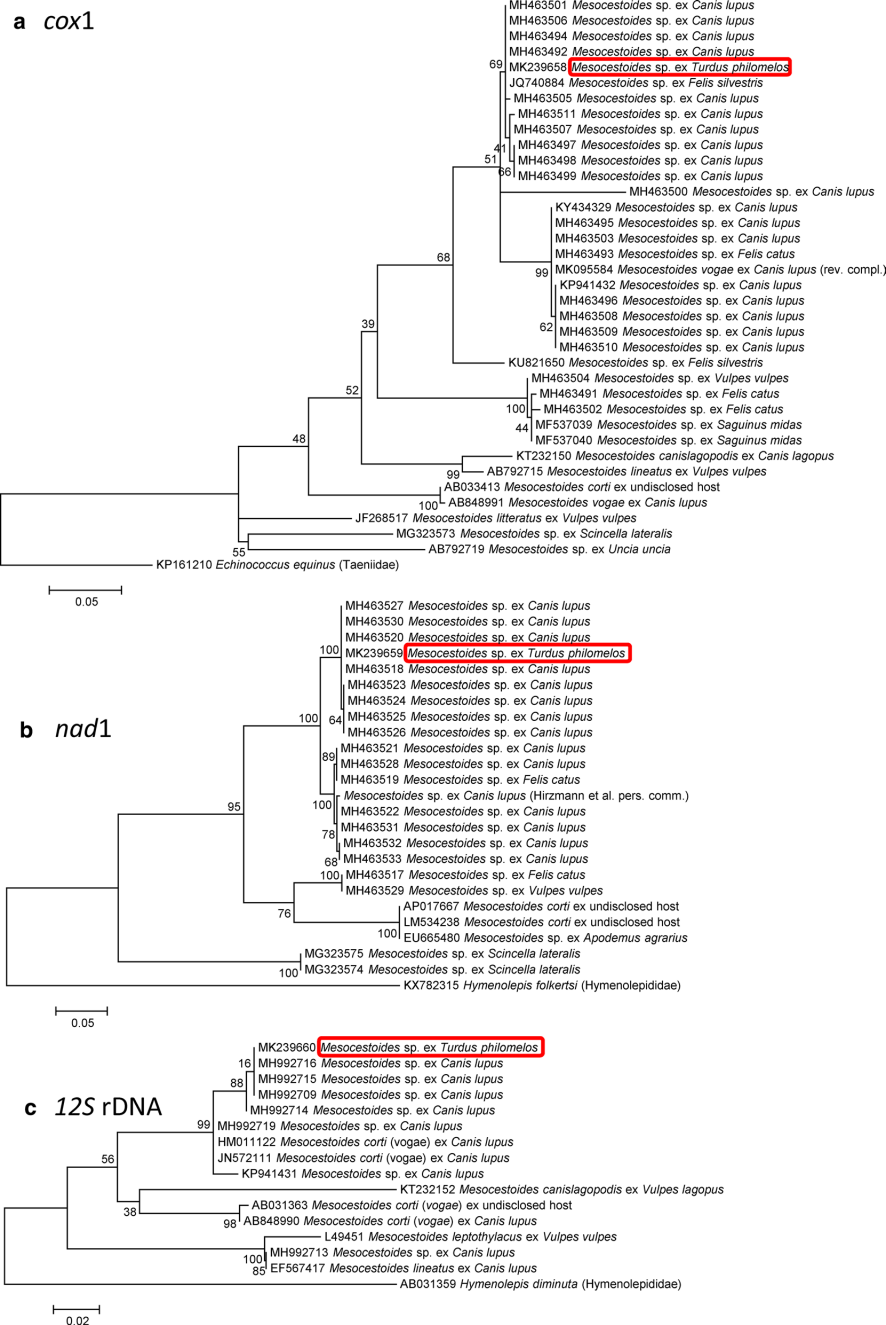
Maximum likelihood analyses of the sequences of mitochondrial DNA loci of *Mesocestoides* spp.: **a** *cox*1; **b** *nad*1; and **c** *12S* rDNA. The scale-bars indicate the number of substitutions per nucleotide site

**Fig. 3:**
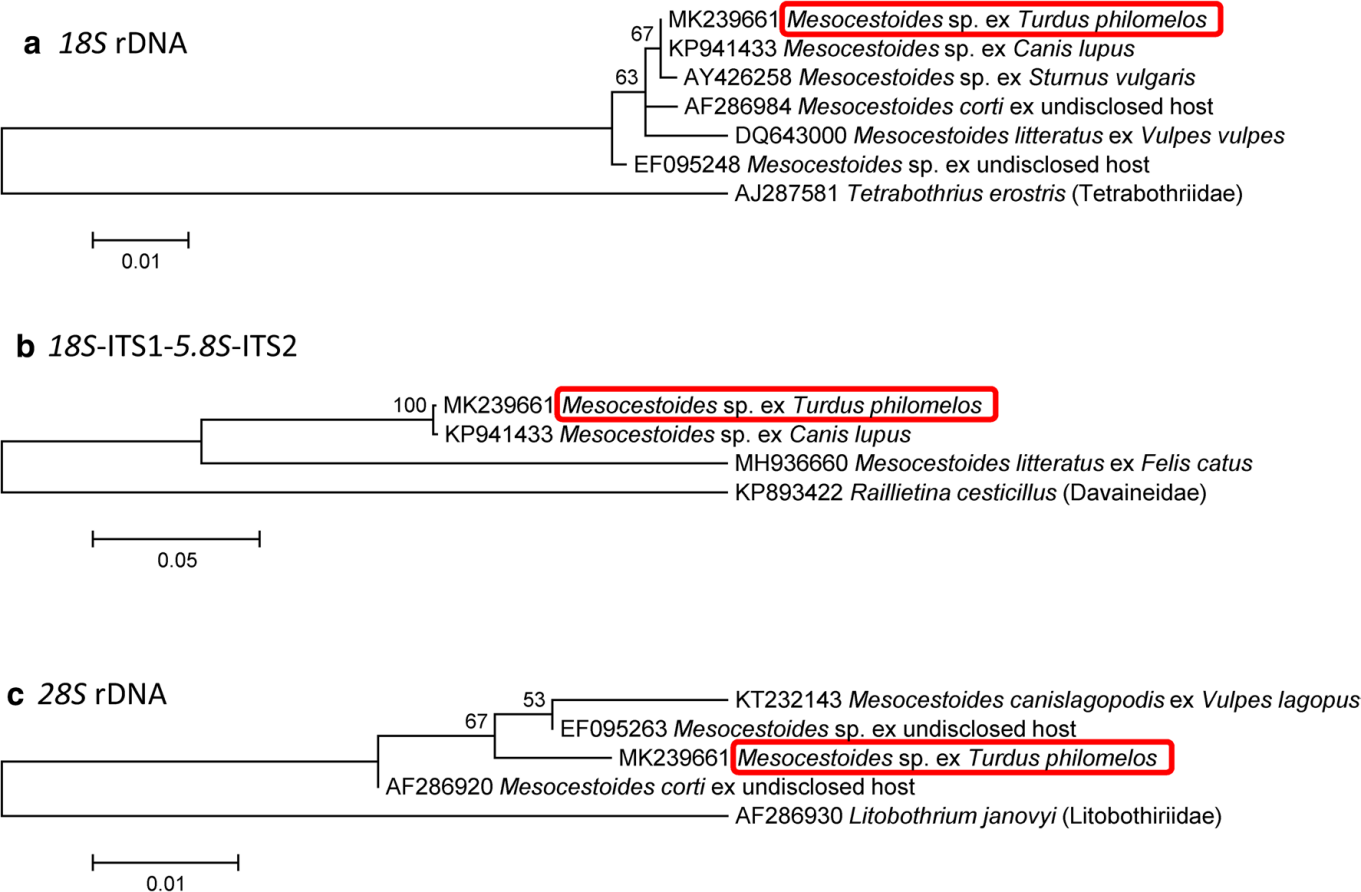
Maximum likelihood analyses of the sequences of nuclear ribosomal DNA loci of *Mesocestoides* spp.: **a** *18S* rDNA; **b** 3′ part of *18S*-ITS1–*5.8S*-ITS2; and **c** *28S* rDNA. The bars indicate the number of substitutions per nucleotide site

The molecular analyses revealed that the examined species was nested within other *Mesocestoides* spp. but was not identical with any *Mesocestoides* spp. previously identified at the species level and subjected to genetic analyses (Figs. 2, 3, Additional file 1: Figure S1, Additional file 2: Figure S2, Additional file 3: Figure S3, Additional file 4: Figure S4, Additional file 5: Figure S5, Additional file 6: Figure S6). The *12S* rDNA locus sequence divergences of *M. lineatus*, *M. canislagopodis*, *M. corti*/*M. vogae* and *M. leptothylacus* from the analyzed species were 10.7 ± 2.3%, 12.8 ± 2.9%, 4.3 ± 1.1% and 12.7 ± 2.6%, respectively (Additional file 7: Table S1). *12S* rDNA was unavailable for *M. litteratus*; this species has a divergence of 15.0 ± 2.6% in the *cox*1 locus, the sequence divergence of which reached 13.2–15.1% for other examined *Mesocestoides* spp. that were identified to species. The *18S* rDNA sequence of the previous genetically characterized isolate from birds, *M. litteratus* (GenBank: AY426258, [17]), was similar to the corresponding segment of nuclear rDNA (divergence only 0.2 ± 0.2%). However, *18S* rDNA is not considered a hypervariable marker and other previously sequenced *Mesocestoides* spp. had just slightly higher divergence in this locus; these included *M. corti* (GenBank: AF286984 [38]) (with a divergence of 0.3 ± 0.3%) and *M. litteratus* (GenBank: DQ643000 [40]) (with a divergence of 1.0 ± 0.4%). The *12S* rDNA of the analyzed species was also highly similar to those from larvae that were previously isolated from life-threatening infections of dogs in Turkey and identified as *M. corti* HM011122 [41], with a divergence of 0.7 ± 0.5%, and JN572111 [42], with a divergence of 0.7 ± 0.9%. However, these tetrathyridia clearly do not represent *M. corti* as other individuals, identified as *M. corti* by Kashiide et al. (GenBank: AB848990 [25]) and Nakao (GenBank: AB031363; unpubl.) formed a distinct clade (Fig. 2c). Moreover, other loci, for which *M. corti* was sequenced, did not agree with the identification of the present material as *M. corti* (Figs. 2, 3).

Importantly, besides the above-described associations, the analyzed species was highly similar to or identical with numerous *Mesocestoides* larvae that were not identified at the species level and that were recently sequenced from domestic dogs and cats. The loci with zero sequence divergence included the *cox*1 locus of the larva from a domestic cat (GenBank: JQ740884 [33]) (Fig. 2a); *12S* rDNA locus of larvae from peritoneal cavities of domestic dogs (GenBank: MH992709, MH992714–MH992716; all unpublished) (Fig. 2c); and *18S*-ITS1–*5.8S-*ITS2 locus of a larva from a domestic dog (GenBank: KP941433 [43]) (Fig. 3a, b). Recently, Varcasia et al. [34] reported a series of findings of larvae of this species from domestic dogs of south Italian, Sicilian and Tunisian origin. In addition to larval isolates, the isolate TUN01 (*cox*1 sequence MH463506), which originated from an intestine of a domestic dog from Tunisia, represented the first and only record of an adult individual that is genetically matched to the individuals examined in the present study. However, the authors did not identify this isolate to species and labeled it instead as the cluster “M3” and did not reveal any details concerning its morphology.

The sequences with high similarity (but not complete identity) included *cox*1 and *nad*1 sequences of another series of domestic dogs and cats from south Italy, Sardinia, Sicily and Tunisia [34]; the *nad*1 sequence of an isolate from a domestic dog (Hirzmann et al. personal communication) (Fig. 2b); and the *12S* rDNA sequence of larvae from domestic dogs (GenBank: MH992719 (unpublished) and KP941431 [43]) (Fig. 2c). The Bayesian inference analysis corroborated all the above conclusions (Additional file 7: Figure S7, Additional file 8: Figure S8, Additional file 9: Figure S9, Additional file 10: Figure S10, Additional file 11: Figure S11, Additional file 12: Figure S12; the colors of the branches indicate Bayesian posterior probabilities).

Combined, the molecular analyses suggest that the presently examined isolate represented a distinct species of *Mesocestoides* that was previously repeatedly isolated as an unidentified larval and adult form from canine and feline body cavities, with numerous DNA sequences available, but with no match to sequences of adult cestodes identified at the species level.

## Discussion

The molecular analyses performed based on the isolate reported in the present study and previously reported isolates from body cavities of domestic dogs and cats suggest the presence of larvae of a species which does not match with sequences of any of the species identified on the basis of adult individuals. Before concluding that this is a new species, we should keep in mind that two further European species of *Mesocestoides*, *Mesocestoides perlatus* (Goeze, 1782) that parasitizes birds of prey and *Mesocestoides melesi* Yanchev & Petrov, 1985 that parasitizes badgers [15, 40] have not been sequenced until now.

The morphology of the above-described metacestodes isolated from *T. philomelos* in Czechia is in agreement with previous descriptions of tetrathyridia of *Mesocestoides* [13–15, 17, 20, 21, 44–46]. The identification of tetrathyridia to the species level based on their morphology is, however, nearly impossible at the present stage [18, 29, 44]. For a more detailed morphological comparison of tetrathyridia, see Literák et al. [17]. However, we believe that morphological documentation of the records is important in view of further comparisons, when more numerous molecular data associated with morphological descriptions have been accumulated.

The isolates from mammals that were genetically similar or identical are described in detail below. Yildiz & Tong [41] reported acephalic metacestodes free on the omental surfaces covering spleen, liver and small intestine of an examined dog, which manifested leukocytosis and an increased hematocrit, but did not provide any morphological details. Their diagnosis of the metacestodes as *M. corti* is here rejected based on the phylogenetic analyses performed in the present study (Fig. 2c). Aypak et al. [42] reported abundant acephalic metacestodes alongside numerous empty calcified cysts of a diameter less than 6 mm that were aggregated into multiple free aggregates of 15 mm in diameter in the peritoneum of an examined dog. The dog manifested normocytic normochromic anemia with low red blood cell count and low hemoglobin, and neutrophilic leukocytosis. Diffuse ascites were present and the peritoneal cysts caused the presence of diffusely scintillating abdominal hyperechoic foci as detected by an X-ray examination. Fibroproliferative peritonitis, chronic gastritis with stomach ulcer and perforation, mycotic pyogranulomatous inflammation of spleen, proximal tubular epithelial degeneration and interstitial plasma cell infiltration of the kidneys and passive hyperemia and fibrosis of the liver were described. Consistent with the above-described case, the larvae did not have distinct scolex and sucker structures that would otherwise be characteristic for the tetrathyridia; the larvae were described as covered by a thick eosinophilic integument. The authors believed that they observed the asexual reproduction of acephalic metacestodes by budding, which is consistent with Galán-Puchades et al. [47], who identified three patterns of asexual reproduction in metacestodes of the genus *Mesocestoides*: longitudinal fission, budding and development of daughter metacestodes in a mother larva. The issue of asexual reproduction of *Mesocestoides* larvae remains controversial, with Specht & Voge [48], Etges [49] and other authors arguing for its presence in laboratory animals. Conn [50] criticized the multiple records of asexually reproducing *Mesocestoides* larvae as doubtful, believing this phenomenon to be well-documented only for a single population of *M. corti* from California studied by Specht & Voge [48]. In the present study, we did not observe any sign of asexual reproduction among the morphologically examined tetrathyridia. Recently, Varcasia et al. [34] reported numerous genetically matched cases from domestic dogs and cats from Italy and Tunisia but did not provide any details on the infection symptoms except of the localization of infection.

Concerning other genetically matched cases of infections, Jabbar et al. [33] reported an infected cat, which displayed severe pan-leucopenia, increased liver enzyme concentrations in blood, namely increased γ-glutamyl transferase and aspartate aminotransferase, and mild azotemia. The animal displayed a right ventricular, hypertrophic cardiomyopathy and generalized lung damage, with areas of parenchymal collapse, carnification and discoloration, consistent with chronic respiratory disease. Macroscopic examination of the abdominal cavity and visceral organs did not reveal any parasites but a thoracotomy, by midline sternotomy, detected nine acephalic metacestodes, being 1–5 cm in length in the thoracic cavity. Similarly to above cases, the authors did not provide any additional morphological details. Finally, Häußler et al. [43] reported a dog with a hyperechoic mass in the middle abdomen detected by sonography and X-ray and with a middle grade systemic inflammatory reaction, mild anemia and low albumin and high globulin serum concentrations. Consistently with the three above studies, the larvae were described as lacking scolex and sucker structures, thus they have to be considered acephalic metacestodes (despite the authors calling them tetrathyridia). These larvae were 0.5–1.5 cm in length, with no further morphological details being provided.

Combined, four of the five previous studies that identified the examined species in mammals reported the agent as the acephalic metacestode stage. It remains unclear, however, if these were metacestodes in the process of development, at the stage before differentiation of the scolex and neck, or if these are developed individuals with an invaginated scolex and neck, which can be visualized by application of staining and/or clearing techniques (Fig. 1). Tetrathyridia, and even an adult individual of the genetically matched individuals, were recently reported from Italy and Tunisia [34]. The study characterized the species using the mitochondrial DNA; however, it did not reveal any details concerning the morphology of tetrathyridia and even of the examined adult individual.

The genetic correspondence of the tetrathyridia found by us with adult worms isolated from the intestines of a Tunisian dog is not surprising in view of the migration of the avian host. The song thrush (*Turdus philomelos*) is a breeding bird in Central Europe. The bird examined by us in the very early spring was obviously found soon after its spring migration. Based on recoveries, more than half of birds ringed in their nesting areas in central Europe were recognized as wintering in Maghreb [51].

## Conclusions

In the present study, we describe a rare and likely lethal case of an infection of a songbird by *Mesocestoides* sp. tetrathyridia. Phylogenetic analyses revealed that the examined isolates are genetically identical or similar to isolates from domestic dogs and cats reported across the Western Palearctic. Four of the five previous studies described infection by the acephalic metacestode stage only; the fifth study, which reported tetrathyridia and an adult individual, did not reveal any morphological details. The possible identity with species previously reported to infect birds remains uncertain. The newly provided DNA sequences should allow the assignment to species in future when adults of the genus *Mesocestoides* are more thoroughly sequenced.

## Methods

Necropsy of a free-ranging female of *Turdus philomelos*, found dead in the vicinity of Záhlinice (eastern Czechia, 49°16′N, 17°28′E) on April 9, 2018, revealed the presence of a large number of tetrathyridia in cysts that were localized across the whole body cavity. For the identification of parasites, we collected all the tetrathyridia and fixed them in 96% ethanol for further analyses. For the comparative morphological analyses, we stained a set of 36 individuals with iron acetocarmine as described by Georgiev et al. [52], dehydrated in an ascending ethanol series (70, 80, 90, 96 and 100%), cleared in dimethyl phthalate and mounted in Canada balsam. Their metrical data are presented as the range followed (in parentheses) by the mean ± standard deviation (SD) and the number of measurements taken (*n*). All measurements are provided in μm. Voucher specimens are deposited in the Invertebrate Collection of the Natural History Museum of Geneva (four slides, 16 specimens), acquisition no. MHNG-PLAT-121610, and in the Helminthological Collection of the Institute of Biodiversity and Ecosystem Research, Bulgarian Academy of Sciences, Sofia (five slides, 20 specimens), acquisition no. IBER-BAS-C-0159.1.6–1.10.

For molecular examination, we extracted, amplified and sequenced the DNA using the primers that targeted the nuclear ribosomal DNA (*18S*-ITS1–*5.8S-*ITS2 and *28S* rDNA) and three mitochondrial loci (*cox*1, *nad*1 and *12S* rDNA) as described [53]. The primers used are specified in Additional file 14: Table S2. The resulting consensus sequences were submitted to the GenBank database under the accession numbers MK239658 (*cox*1), MK239659 (*nad*1), MK239660 (*12S* rDNA) and MK239661 (nuclear ribosomal DNA).

We aligned the newly generated sequences, sequences of *Mesocestoides* spp. obtained from GenBank as of November 30, 2018, and sequences of corresponding outgroups using ClustalW in MEGA5 (gap opening penalty 7 and gap extension penalty 2 for both pairwise and multiple alignments, DNA weight matrix IUB, and transition weight 0.1). We manually checked and corrected the inconsistencies. We trimmed each alignment to the length of the shortest included sequence. Because the *Mesocestoides* spp. sequences that were available in GenBank did not cover the whole range of the nuclear rDNA sequenced in the present study, we performed three separate alignments of parts of the nuclear rDNA that corresponded to partial *18S* rDNA, the ITS1-*5.8S*-ITS2 segment and the *28S* rDNA. The trimmed *cox*1 locus (partial *cox*1 coding sequence) corresponded to nt. 13–369 (357 bp) of *M. lineatus* AB792715 (Additional file 1: Figure S1). The trimmed *nad*1 locus (partial *nad*1 coding sequence) corresponded to nt. 5309–5720 (412 bp) of *M. corti* AP017667 (Additional file 2: Figure S2). The trimmed *12S* rDNA locus (partial *12S* ribosomal RNA coding sequence) corresponded to nt. 7–299 (293 bp) of *M. corti* AB031363 (Additional file 3: Figure S3). The trimmed *18S* rDNA locus [partial small subunit rRNA coding sequence] corresponded to nt. 370–986 (617 bp) of *Mesocestoides* sp. AY426258 (Additional file 4: Figure S4). The trimmed *18S*-ITS1–*5.8S-*ITS2 locus (partial *18S* rRNA coding sequence, full-length ITS1, full-length *5.8S* ribosomal RNA coding sequence and partial ITS2 sequences) corresponded to nt. 1459–2689 (1231 bp) of *Mesocestoides* sp. KP941433 (Additional file 5: Figure S5). The trimmed *28S* rDNA locus [partial large subunit (*LSU*) rRNA coding sequence] corresponded to nt. 49–301 (253 bp) of *M. canislagopodis* KT232143 (Additional file 6: Figure S6). We analyzed the maximum likelihood fits of the 24 nucleotide substitution models in MEGA5. The best-fitting models according to the Bayesian information criterion were GTR + I (for *cox*1), HKY + I (for *nad*1), HKY + G (for *12S* rDNA), K2 (for *18S* rDNA), HKY (for the ITS1-*5.8S-*ITS2 locus and for the *28S* rDNA locus, respectively). We used bootstrapping at 1000 replicates and the nearest-neighbor-interchange as the maximum likelihood heuristic method of choice to infer the trees and used best-fit models for the maximum likelihood phylogenetic analyses. To analyze the evolutionary divergence of the analyzed species from those that were sequenced previously, we estimated the pairwise distances expressed as a number of base differences per site obtained by averaging over all sequence pairs between groups. The data are shown as the means resulting from the application of the bootstrap procedure with 1000 replicates.

To corroborate the results of maximum likelihood analyses, we performed Bayesian inference analysis in MrBayes v. 3.2.5 using the mixed model of nucleotide substitution and including four Monte Carlo Markov chains for 10,000,000 generations. We discarded the first 25% of the samples as burn-in and used the remaining data to generate a 50% majority-consensus tree with the indicated posterior probabilities of branches. The obtained summary statistics are provided in Additional file 15: Table S3.

## Additional files

**Additional file 1: Figure S1.** Alignment of the trimmed *cox*1 locus (partial *cox*1 coding sequence).

**Additional file 2: Figure S2.** Alignment of the trimmed *nad*1 locus (partial *nad*1 coding sequence).

**Additional file 3: Figure S3.** Alignment of the trimmed *12S* rDNA locus (partial *12S* ribosomal RNA coding sequence).

**Additional file 4: Figure S4.** Alignment of the trimmed *18S* rDNA locus (partial *SSU* rRNA coding sequence).

**Additional file 5: Figure S5.** Alignment of the trimmed *18S*-ITS1–*5.8S*-ITS2 locus (partial *18S* rRNA coding sequence, full-length ITS1, full-length *5.8S* ribosomal RNA coding sequence and partial ITS2 sequences).

**Additional file 5: Figure S6.** Alignment of the trimmed *28S* rDNA locus (partial *LSU* rRNA coding sequence).

**Additional file 7: Table S1.** The *12S* rDNA locus sequence divergences of *M. lineatus*, *M. canislagopodis*, *M. corti*/*vogae*, *M*. *leptothylacus* and *M. litteratus* from the presently analyzed species. Data are shown as mean % of divergence (below the diagonal), with S.E. shown above the diagonal.

**Additional file 8: Figure S7.** Phylogenetic tree based on Bayesian inference of the *cox*1 of *Mesocestoides*.

**Additional file 9: Figure S8.** Phylogenetic tree based on Bayesian inference of the *nad*1 of *Mesocestoides*.

**Additional file 10: Figure S9.** Phylogenetic tree based on Bayesian inference of the *12S* rDNA of *Mesocestoides*.

**Additional file 11: Figure S10.** Phylogenetic tree based on Bayesian inference of the *18S* rDNA of *Mesocestoides*.

**Additional file 12: Figure S11.** Phylogenetic tree based on Bayesian inference of the ITS1-*5.8S* rDNA-ITS2 of *Mesocestoides*.

**Additional file 13: Figure S12.** Phylogenetic tree based on Bayesian inference of the *28S* rDNA of *Mesocestoides*.

**Additional file 14: Table S2.** List of primers that were used to amplify the analyzed DNA loci.

**Additional file 15: Table S3.** Summary statistics for Bayesian inference analyses performed. The data are provided for partitions with frequency ≥ 0.10 in at least one run.

## Data Availability

Voucher specimens have been deposited in the Invertebrate Collection of the Natural History Museum of Geneva (4 slides, 16 specimens), acquisition no. MHNG-PLAT-121610, and in the Helminthological Collection of the Institute of Biodiversity and Ecosystem Research, Bulgarian Academy of Sciences, Sofia (5 slides, 20 specimens), acquisition no. IBER-BAS-C-0159.1.6–1.10. The newly generated consensus DNA sequences were submitted to the GenBank database under the accession numbers MK239658 (*cox*1), MK239659 (*nad*1), MK239660 (*12S* rDNA) and MK239661 (nuclear ribosomal DNA).

## Abbreviations

*cox*1: cytochrome c oxidase, subunit 1
ITS2: internal transcribed spacer 2
*LSU*: large subunit
*n*: number of measurements taken
*nad*1: nicotinamide adenine dinucleotide dehydrogenase, subunit 1
rDNA: ribosomal deoxyribonucleic acid
SD: standard deviation
*SSU*: small subunit
