# Supplementary figures and images for: Massive infection of a song thrush by Mesocestoides sp. (Cestoda) tetrathyridia that genetically match acephalic metacestodes causing lethal peritoneal larval cestodiasis in domesticated mammals
Source: Parasit Vectors. 2019 May 14;12:230. doi: 10.1186/s13071-019-3480-1 (PMC6518502; doi:10.1186/s13071-019-3480-1)

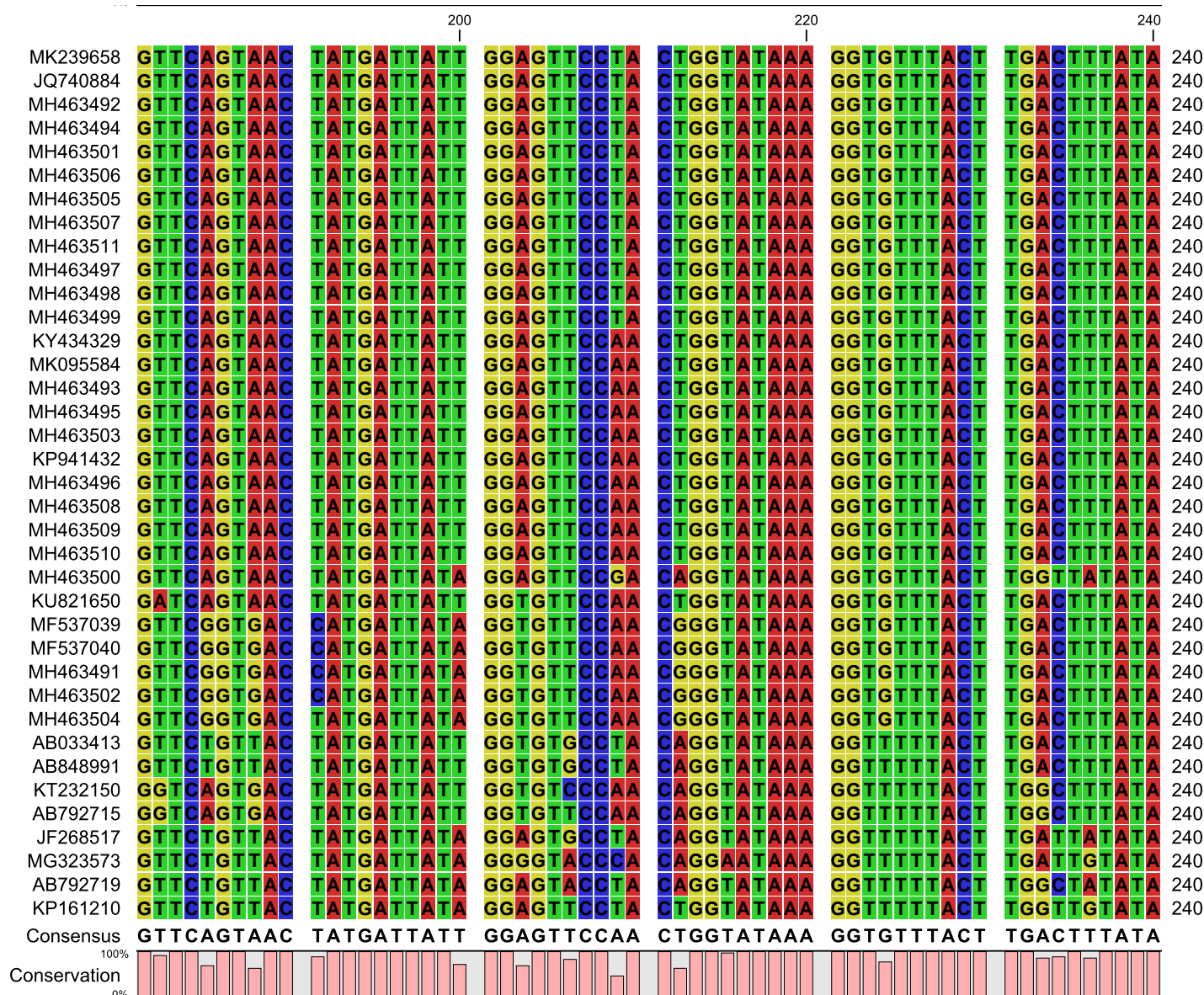

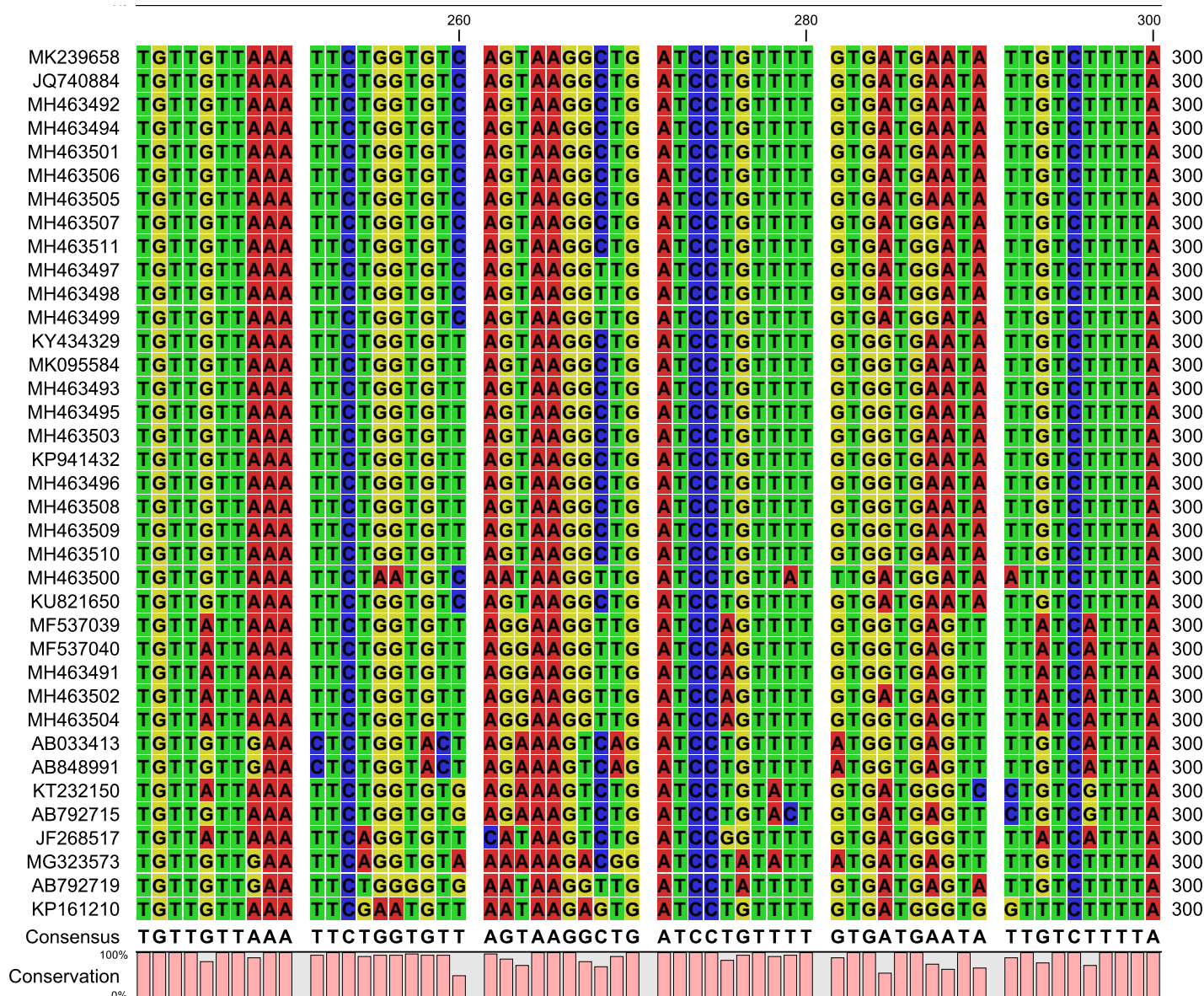

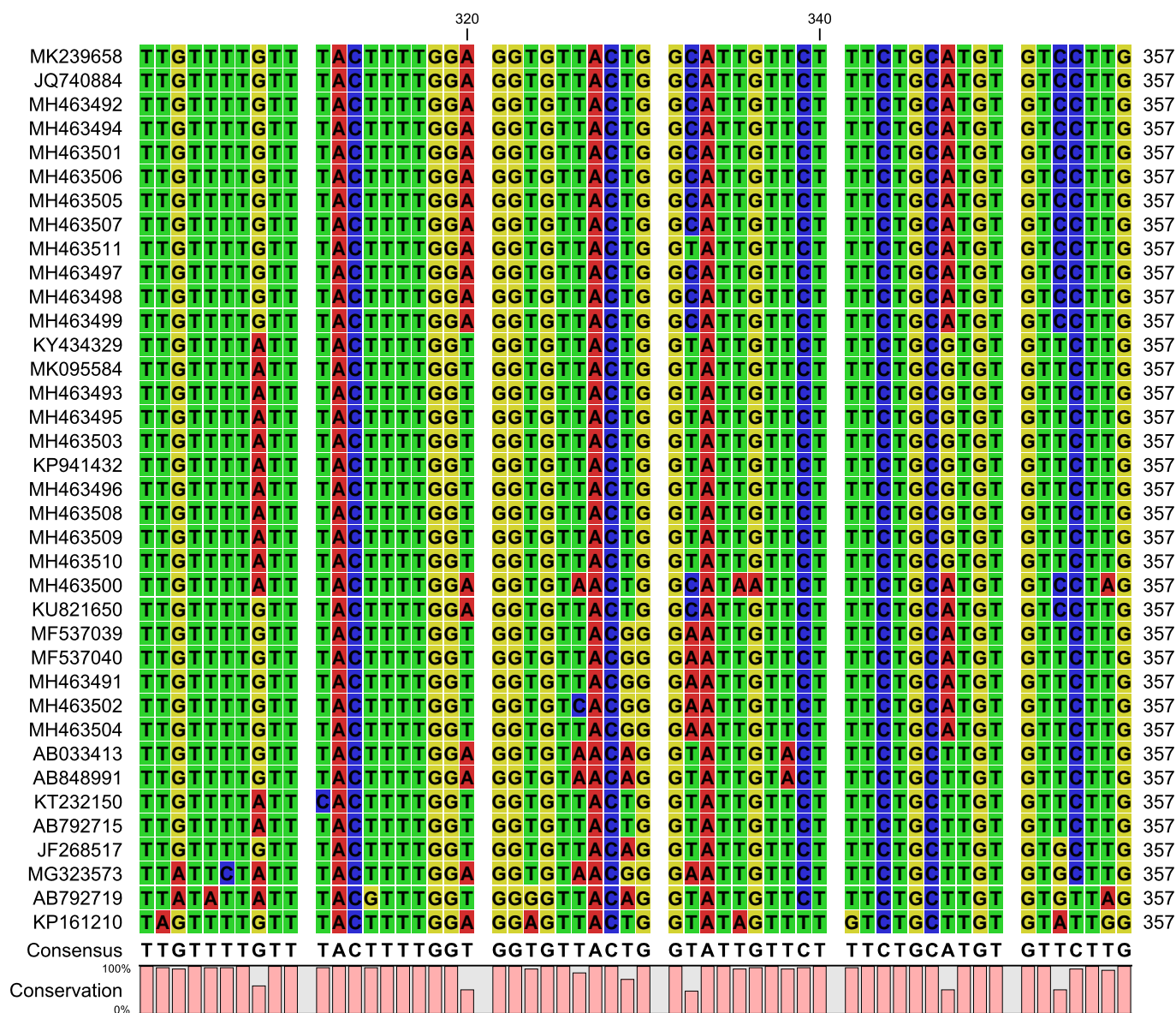

Supplement: Supplementary file 1 — Additional file 1: Figure S1. Alignment of the trimmed cox1 locus (partial cox1 coding sequence). [file 13071_2019_3480_MOESM1_ESM.pdf]

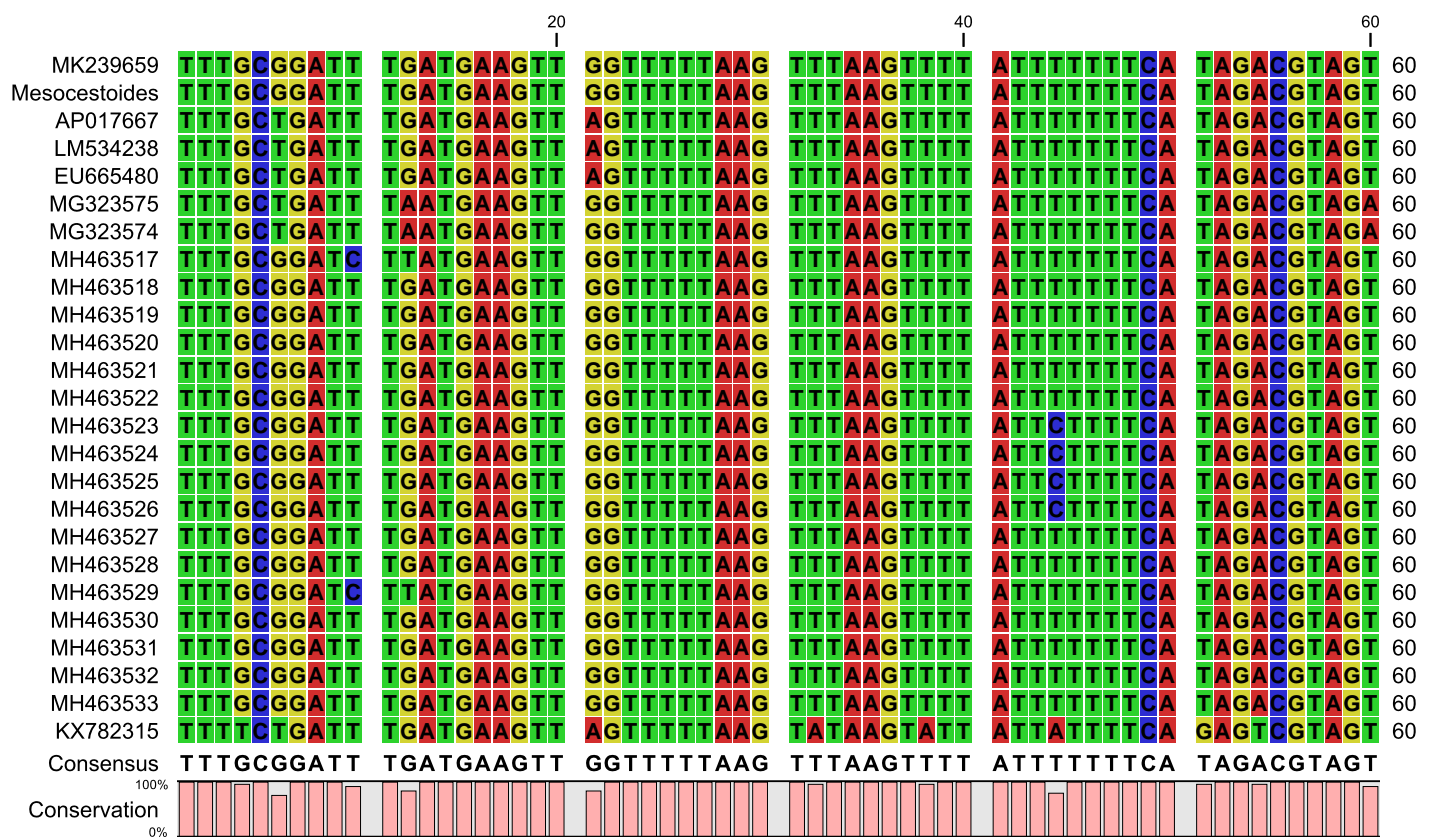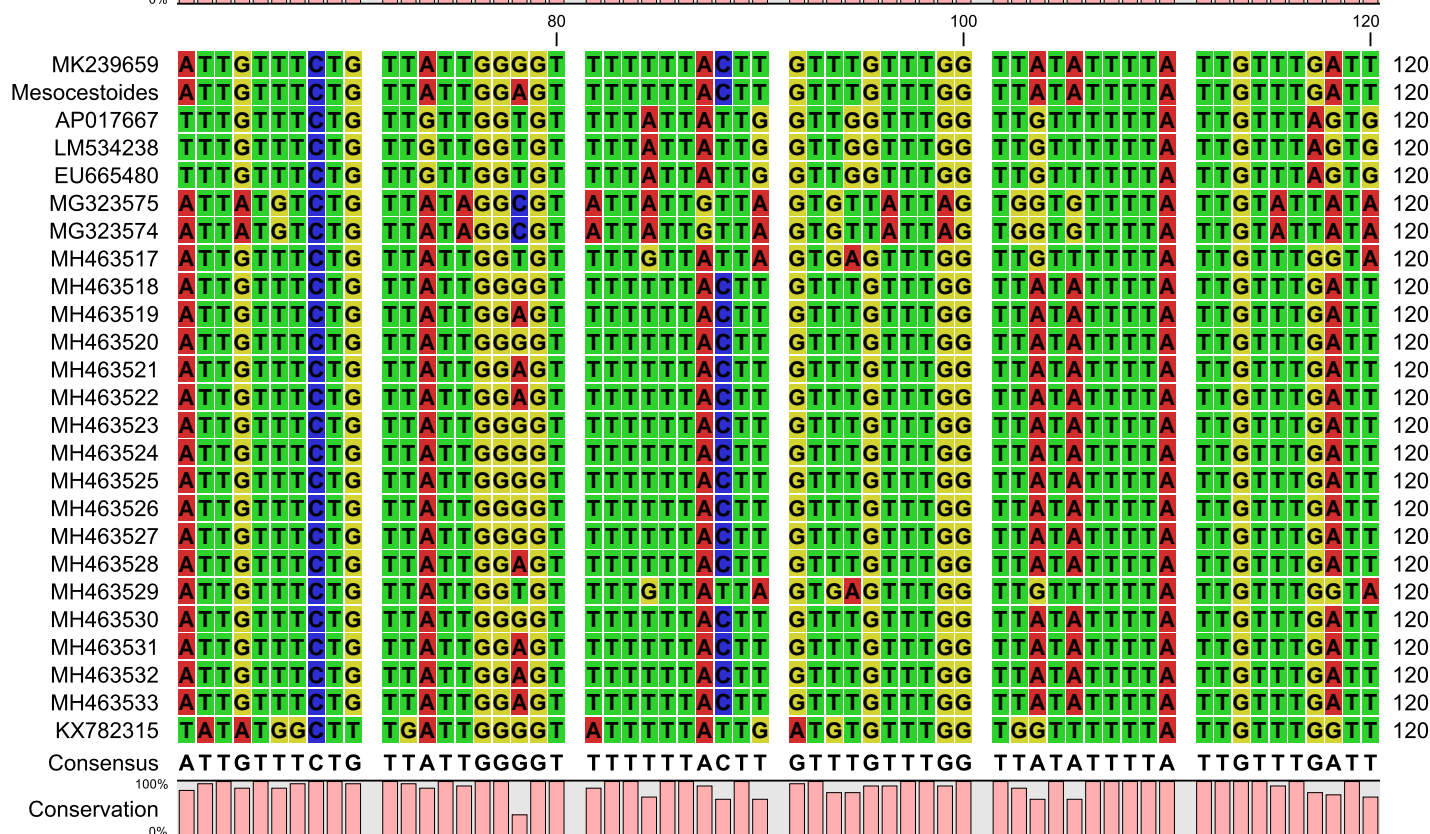

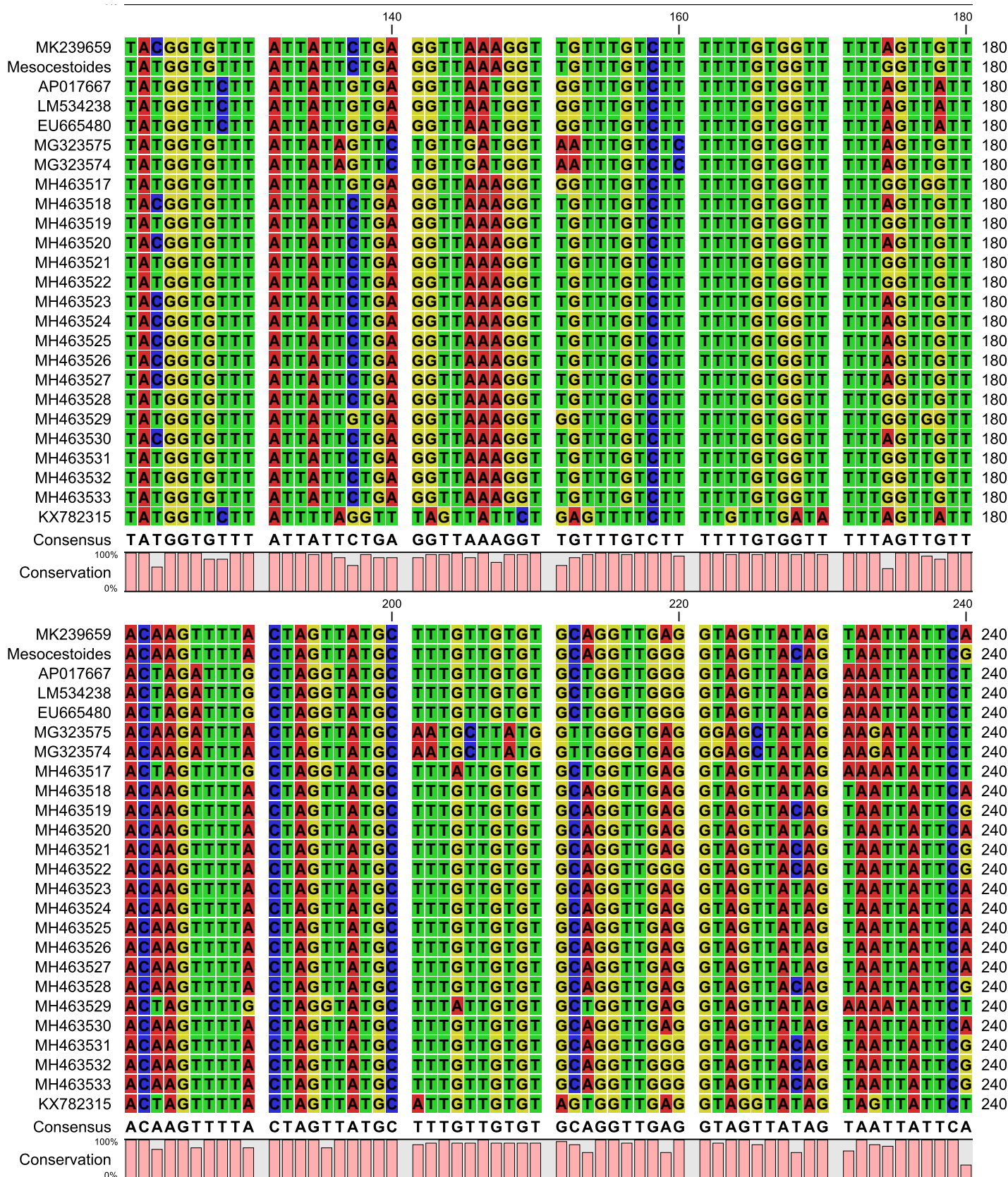

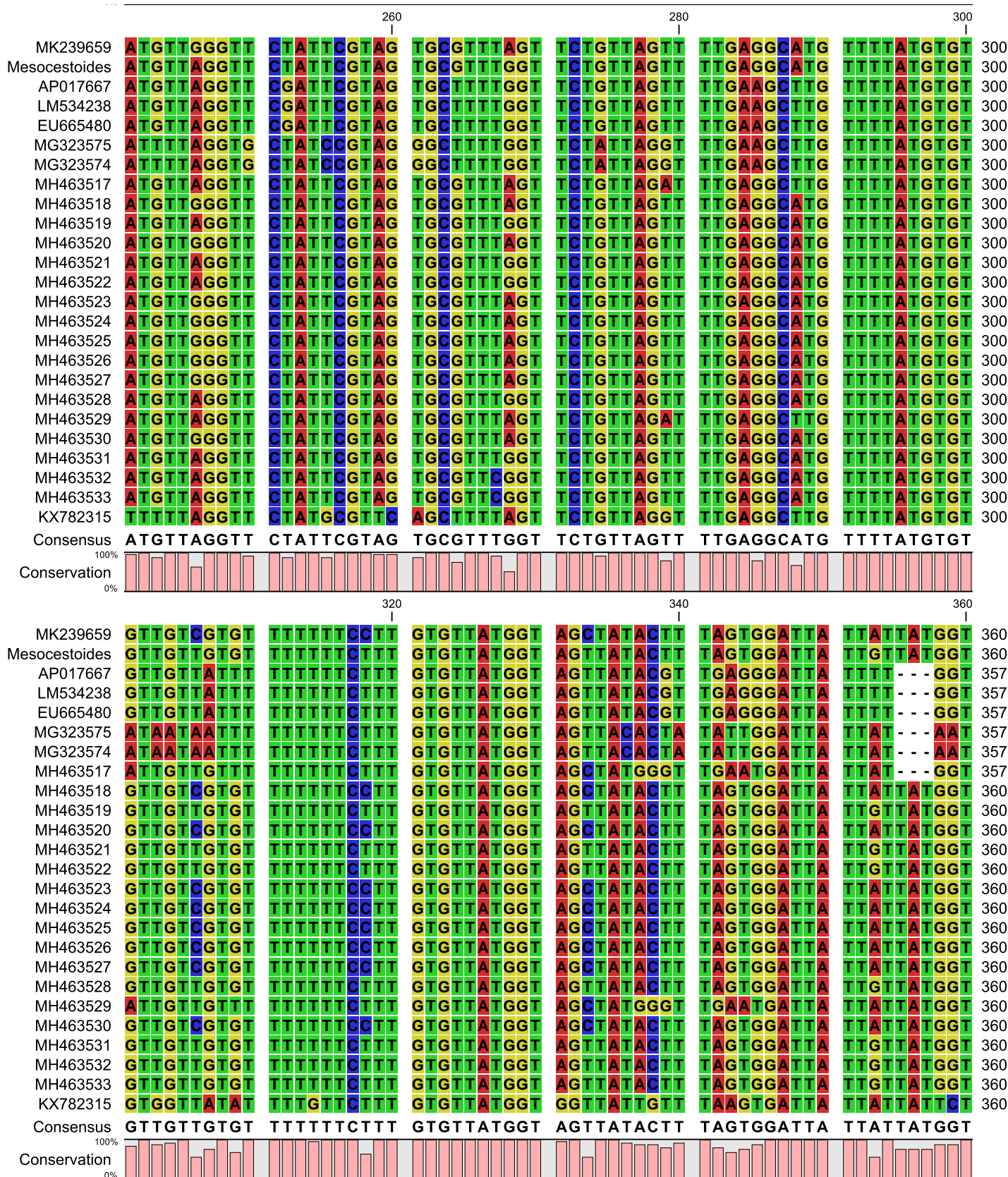

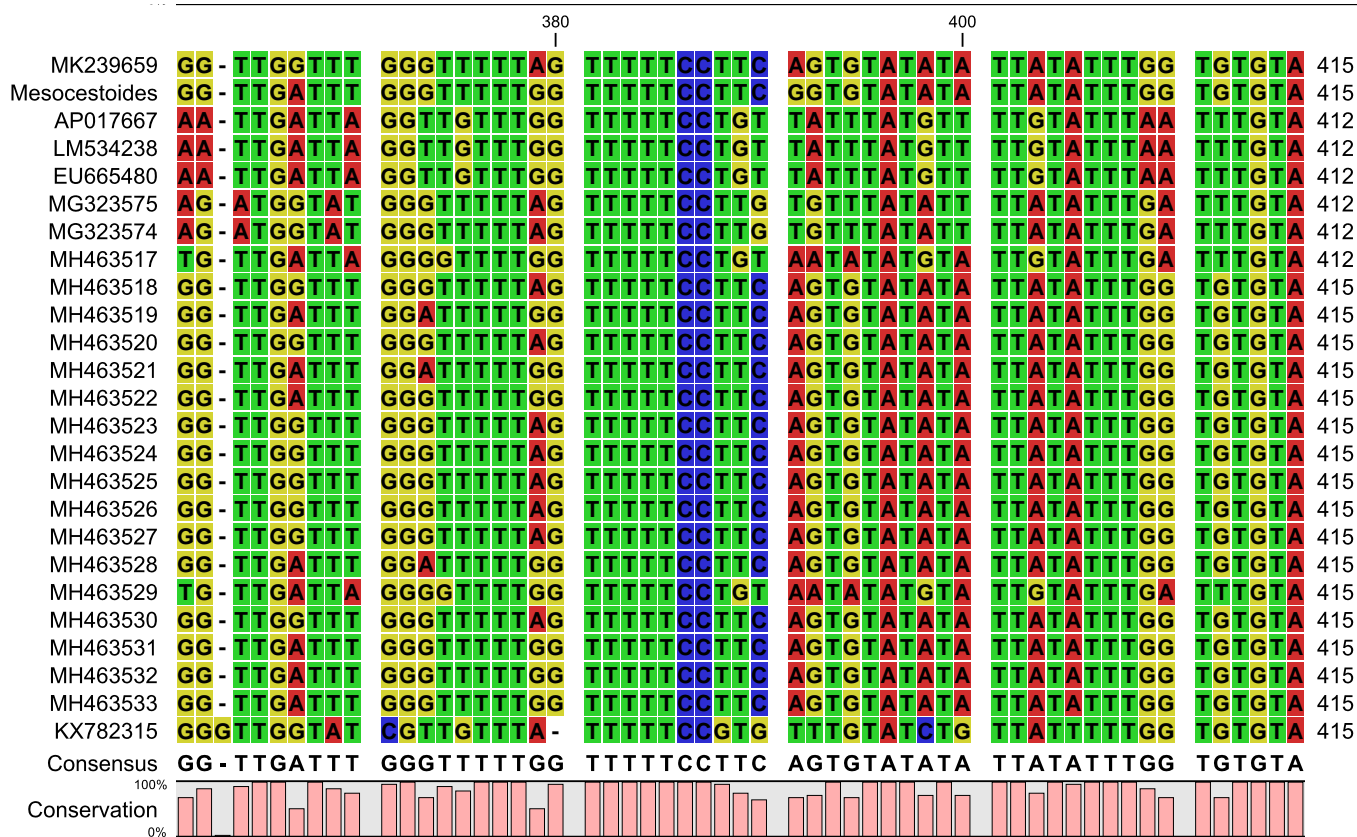

Supplement: Supplementary file 2 — Additional file 2: Figure S2. Alignment of the trimmed nad1 locus (partial nad1 coding sequence). [file 13071_2019_3480_MOESM2_ESM.pdf]

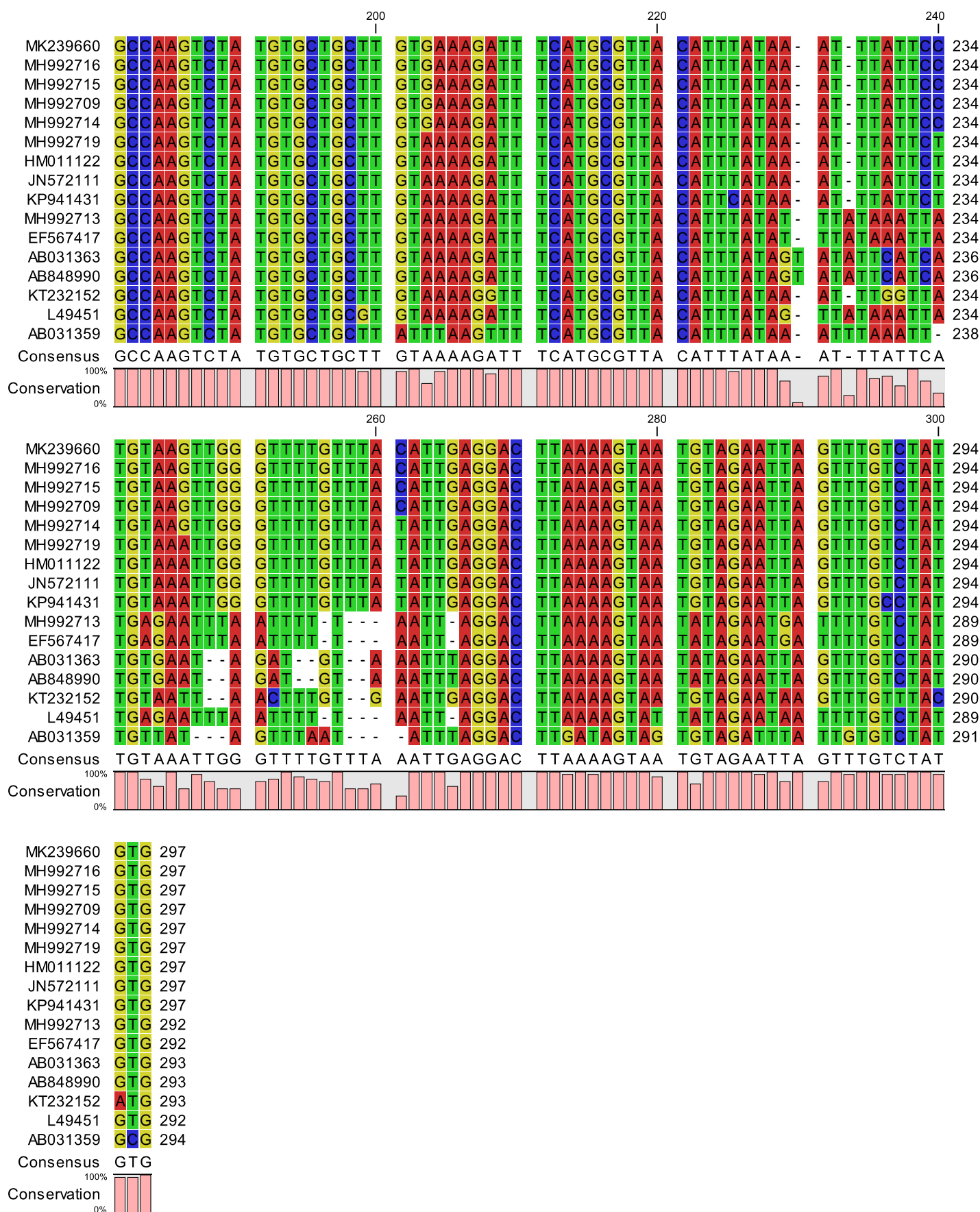

Supplement: Supplementary file 3 — Additional file 3: Figure S3. Alignment of the trimmed 12S rDNA locus (partial 12S ribosomal RNA coding sequence). [file 13071_2019_3480_MOESM3_ESM.pdf]

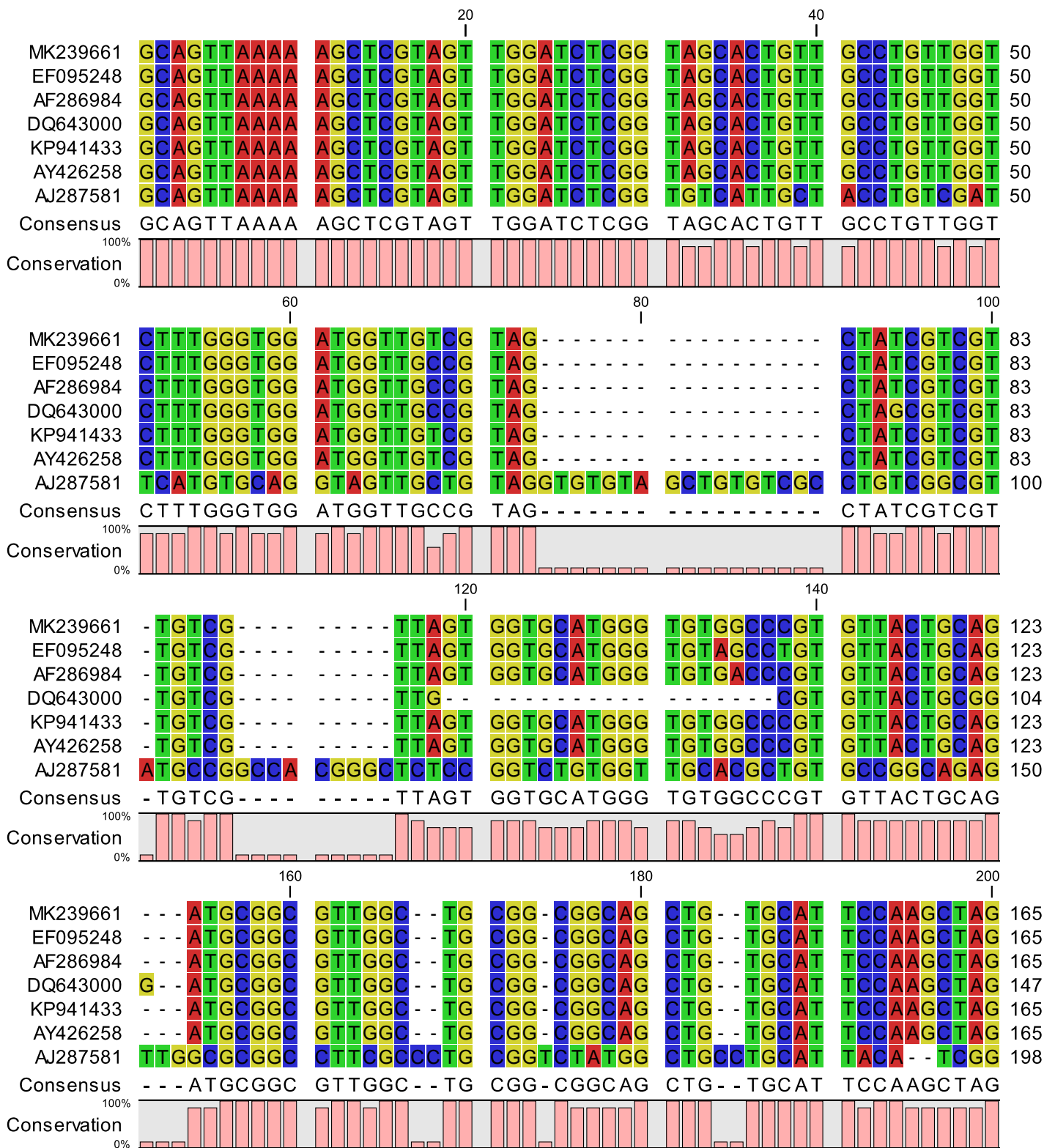

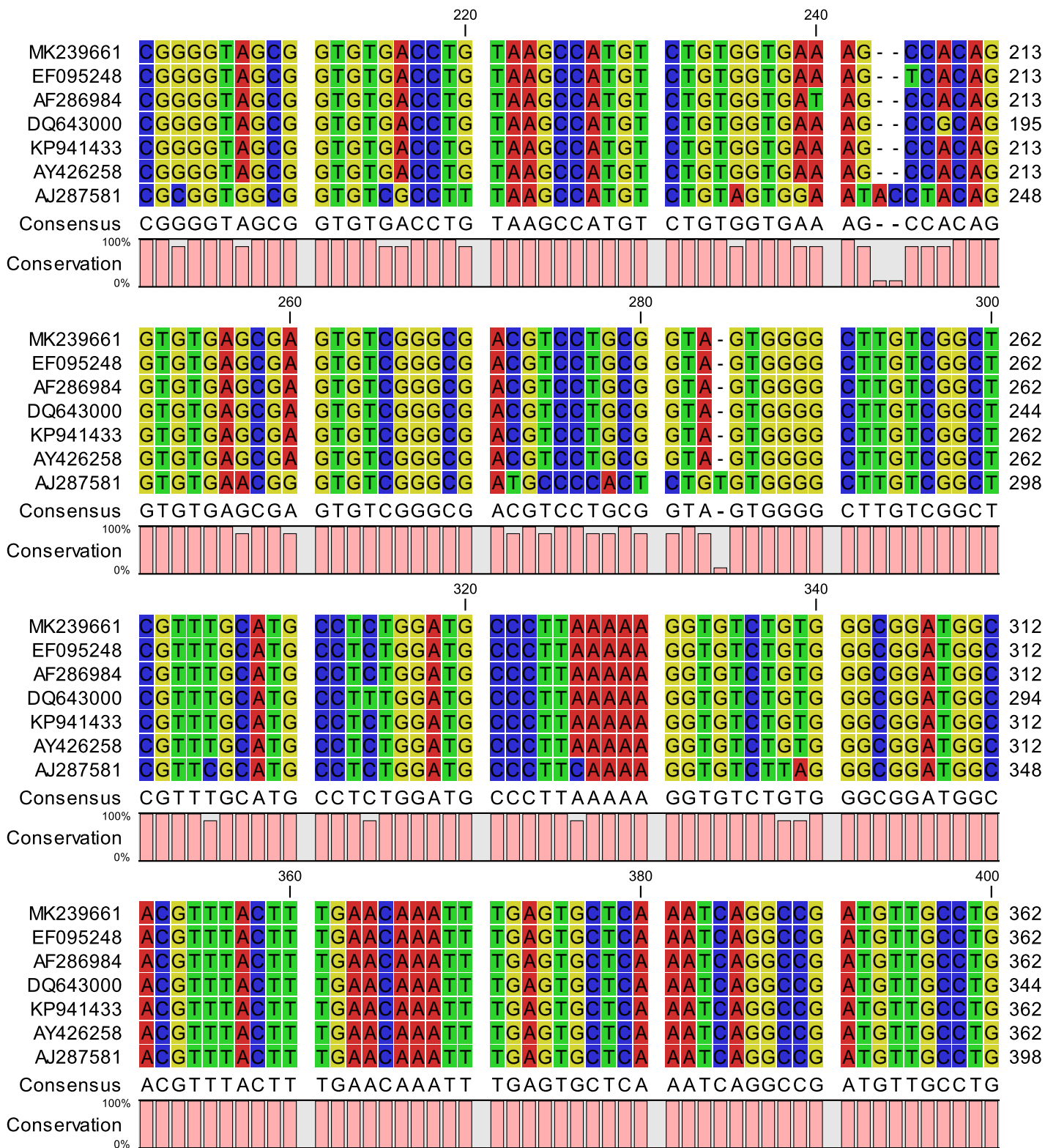

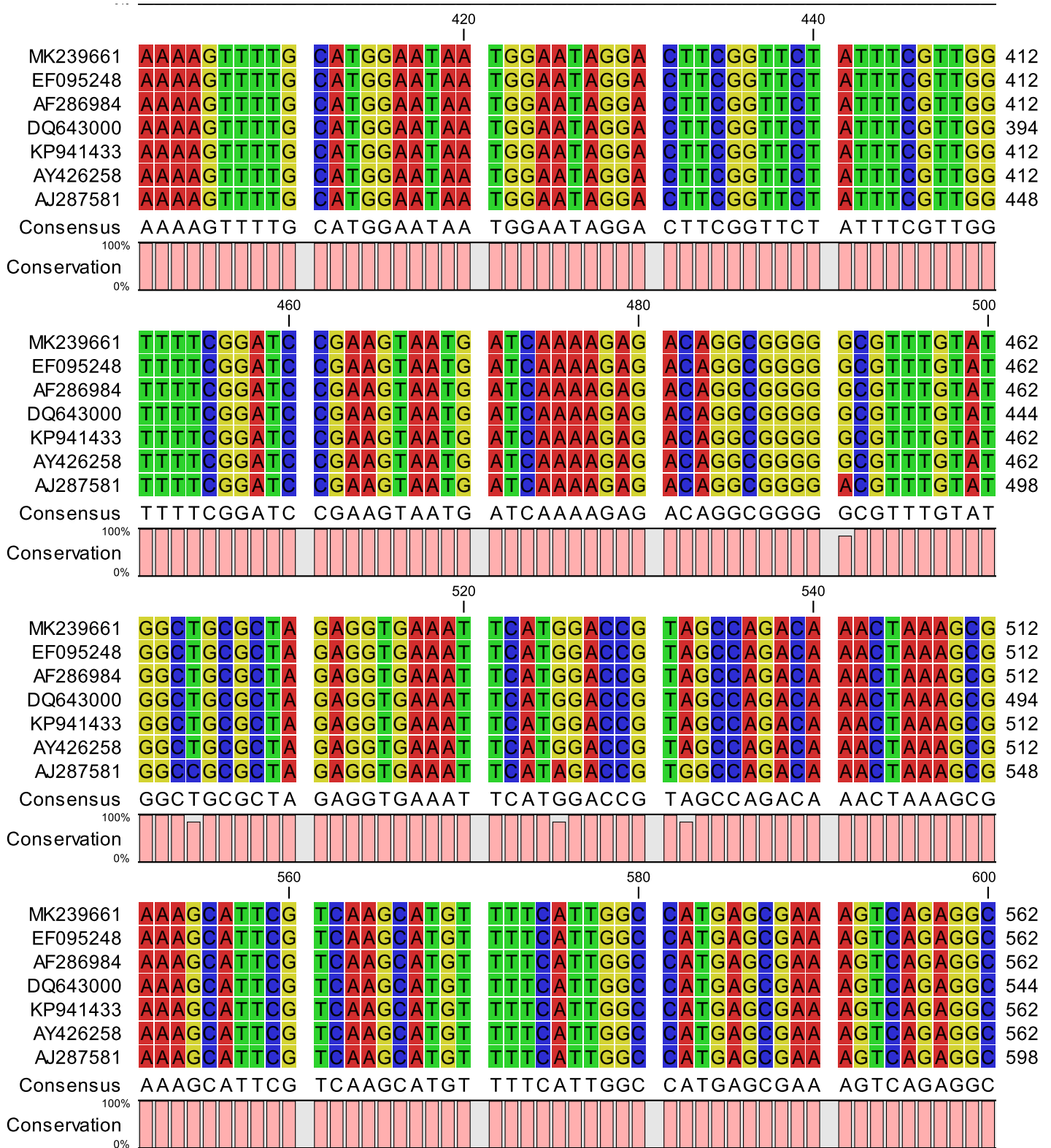

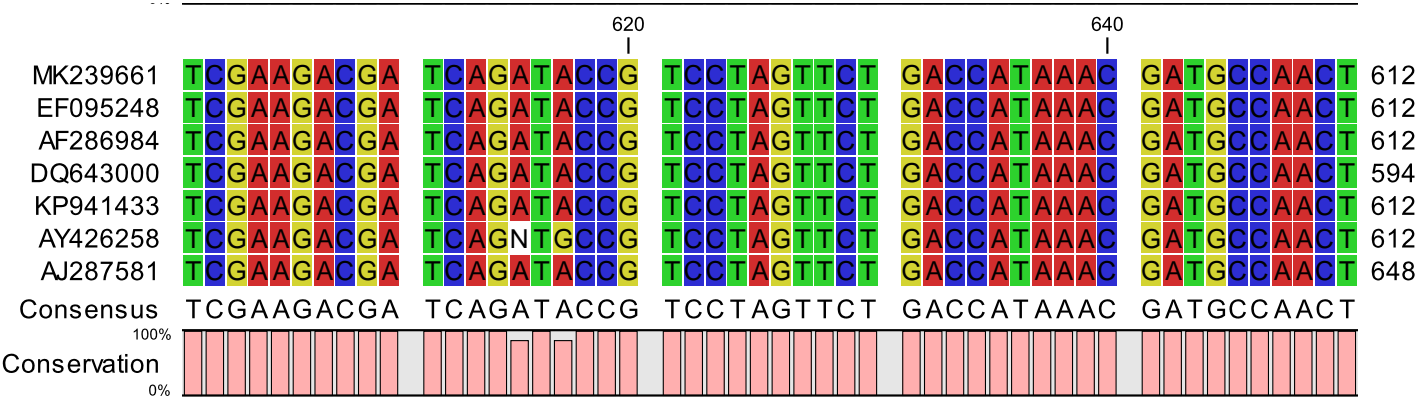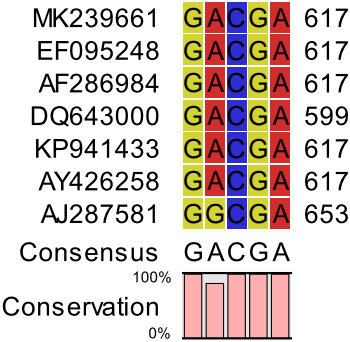

Supplement: Supplementary file 4 — Additional file 4: Figure S4. Alignment of the trimmed 18S rDNA locus (partial SSU rRNA coding sequence). [file 13071_2019_3480_MOESM4_ESM.pdf]

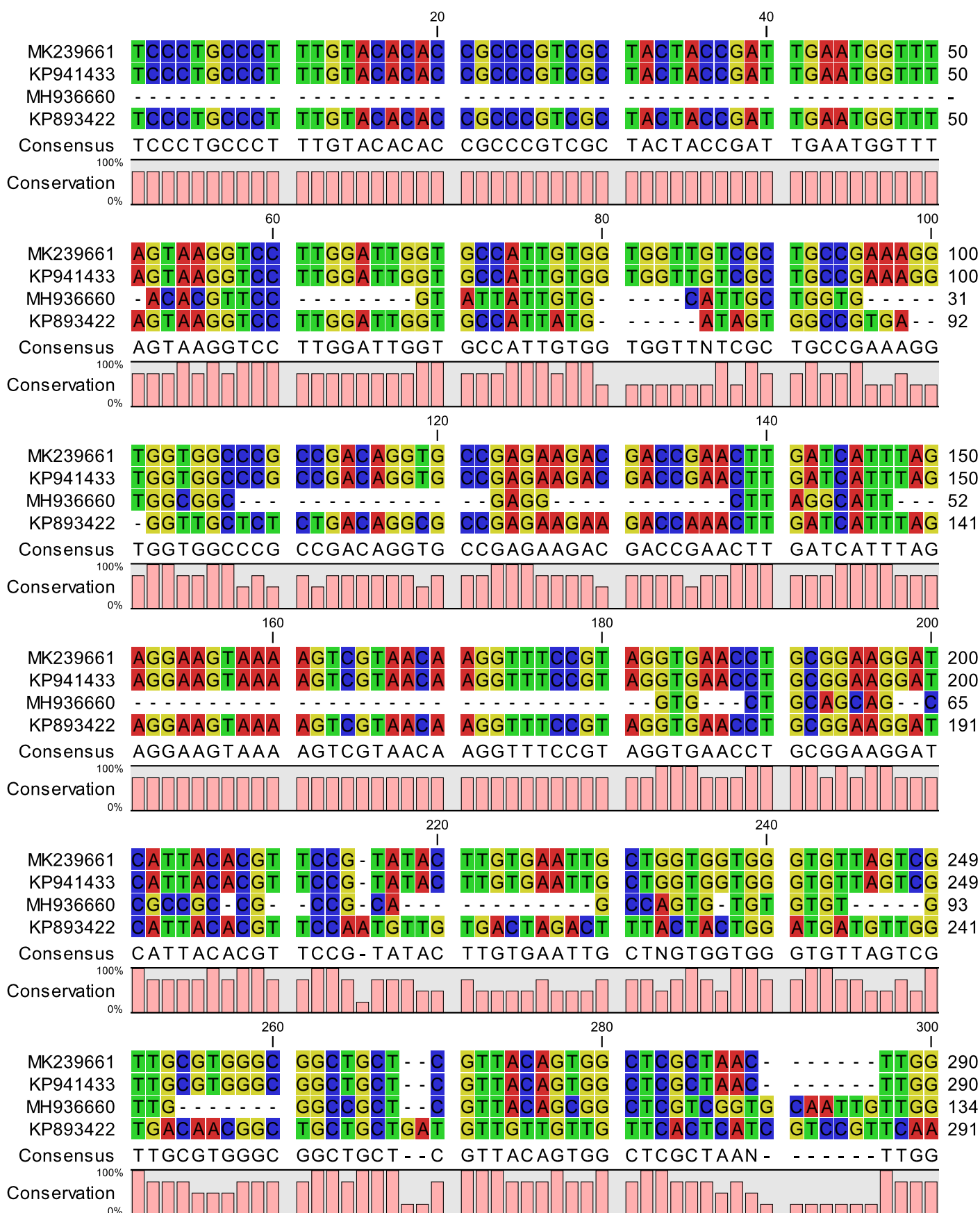

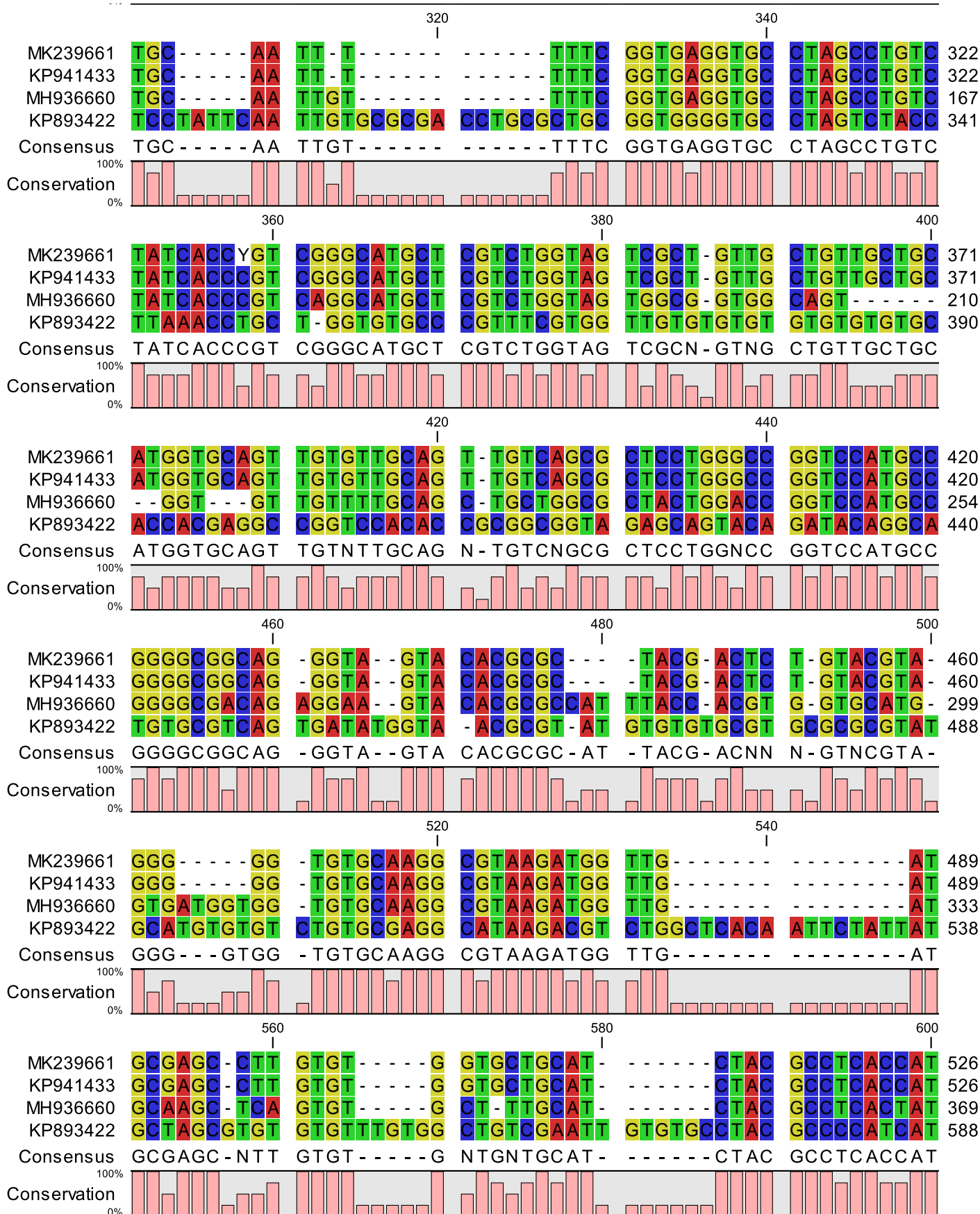

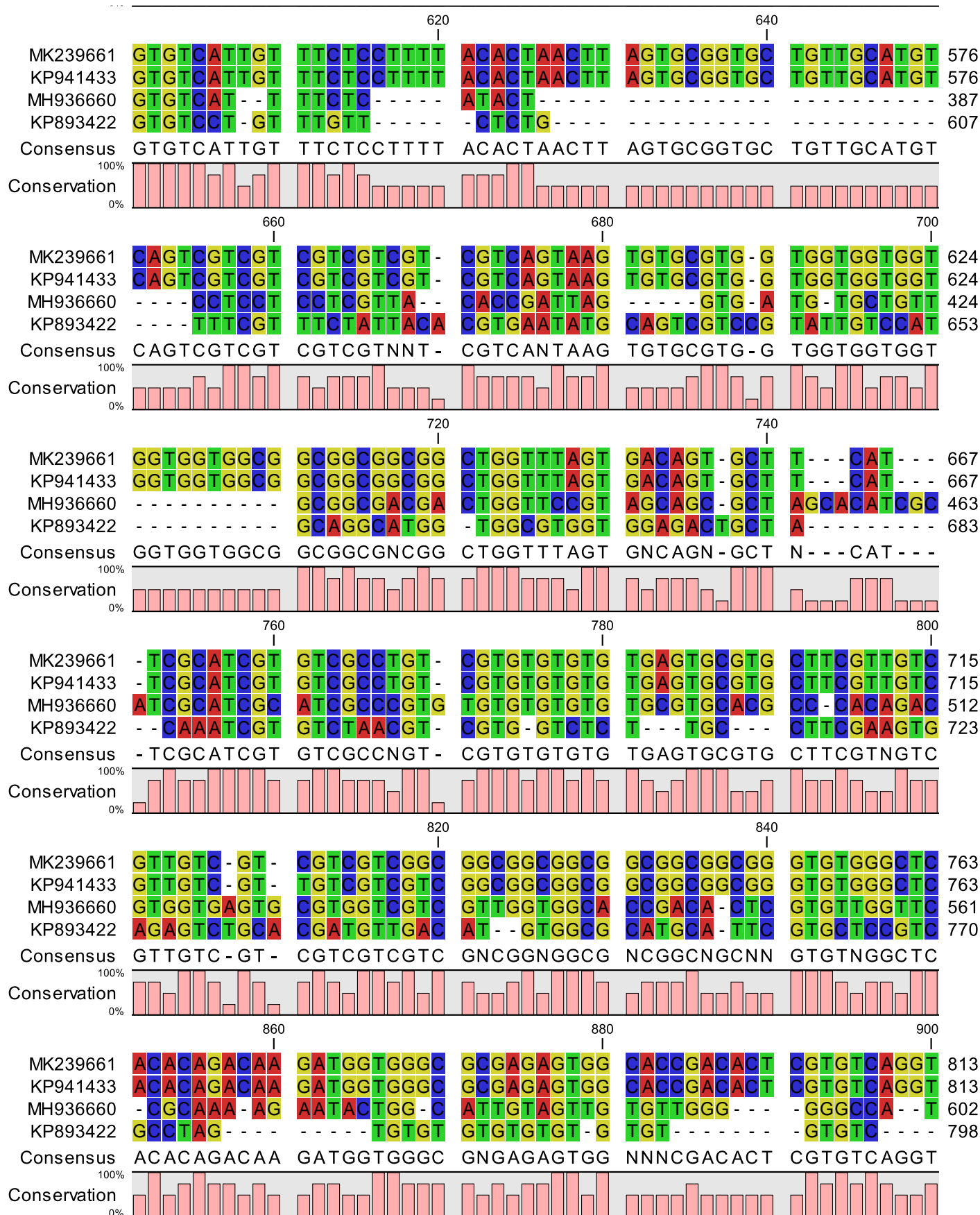

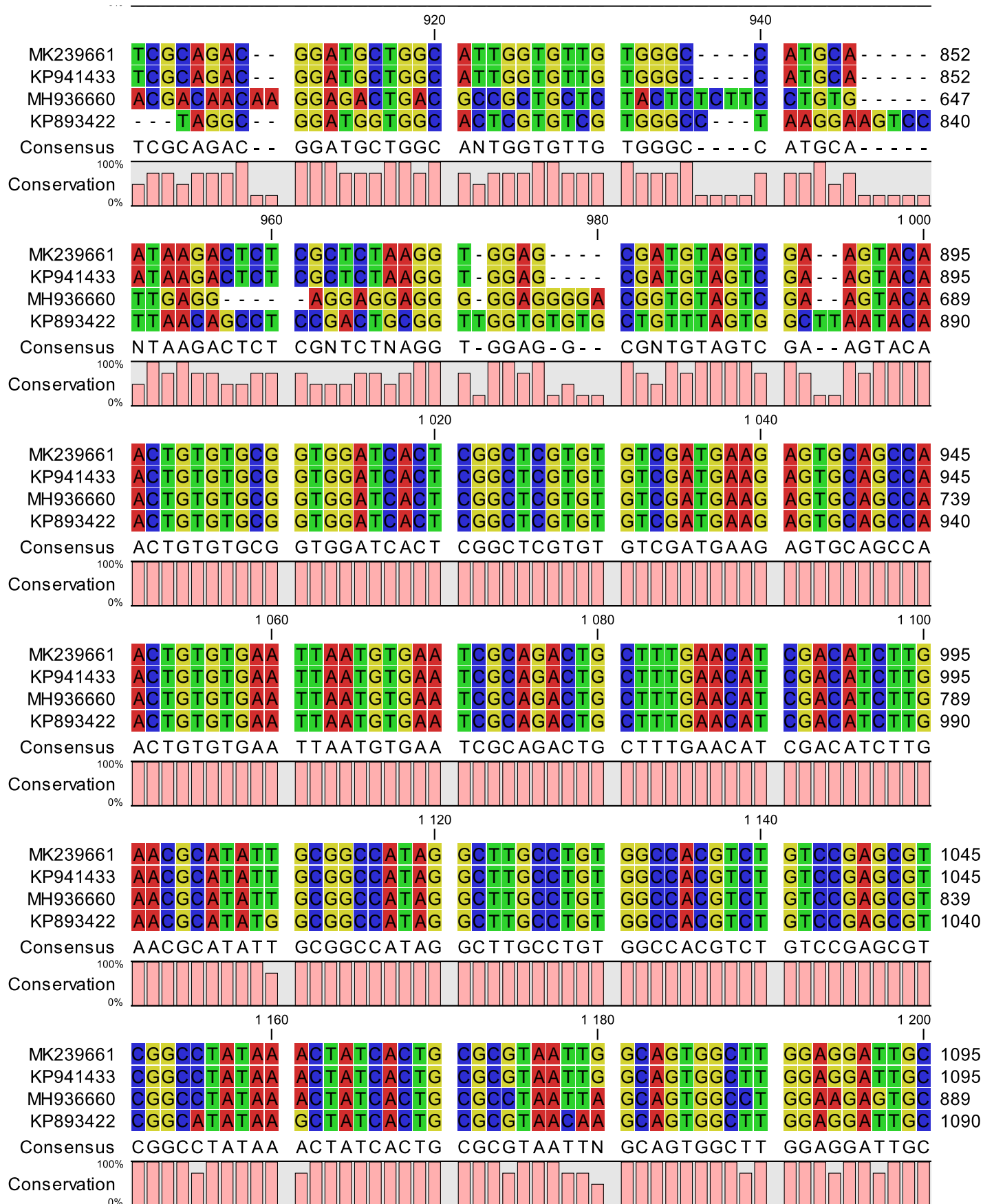

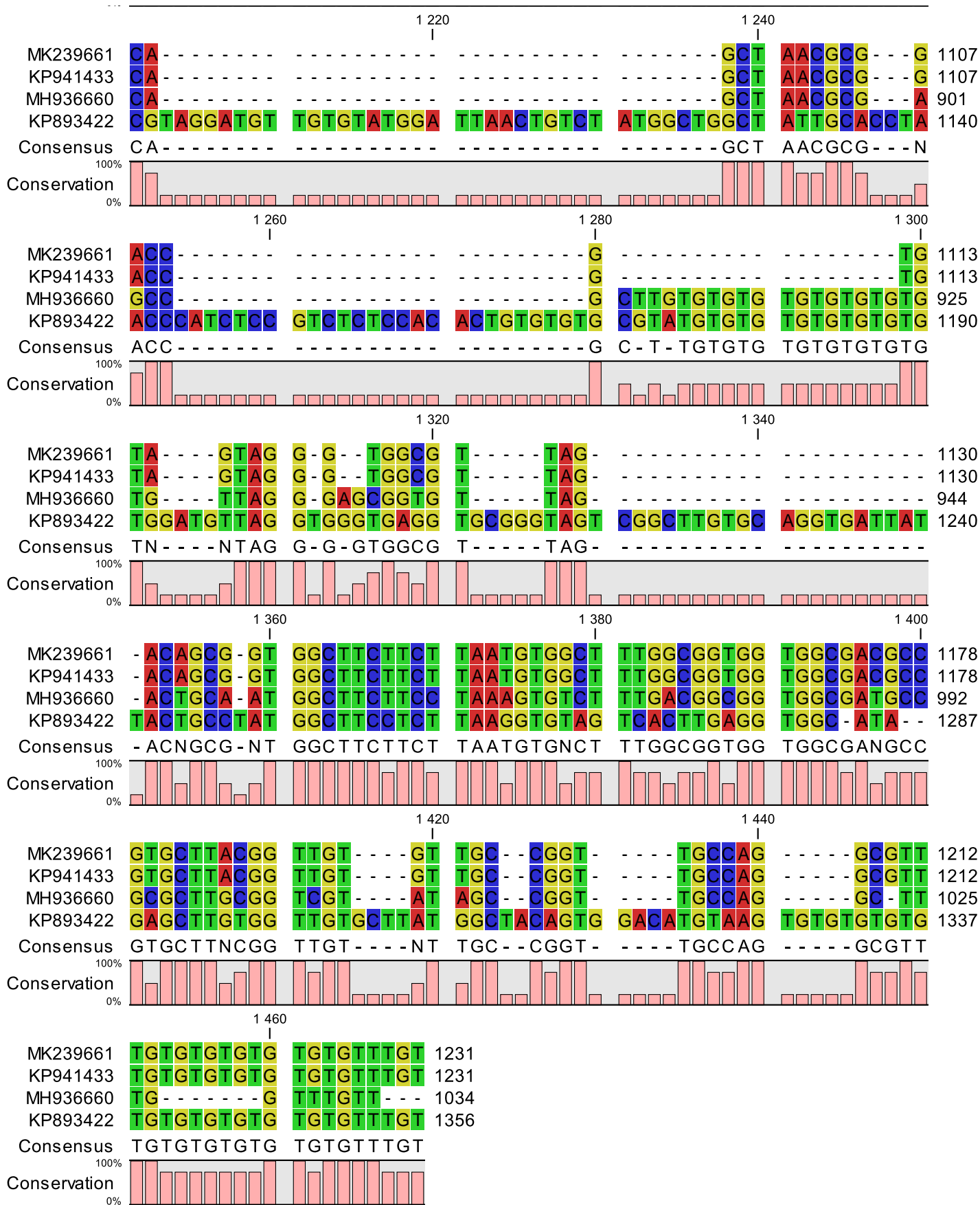

Supplement: Supplementary file 5 — Additional file 5: Figure S5. Alignment of the trimmed 18S-ITS1–5.8S-ITS2 locus (partial 18S rRNA coding sequence, full-length ITS1, full-length 5.8S ribosomal RNA coding sequence and partial ITS2 sequences). [file 13071_2019_3480_MOESM5_ESM.pdf]

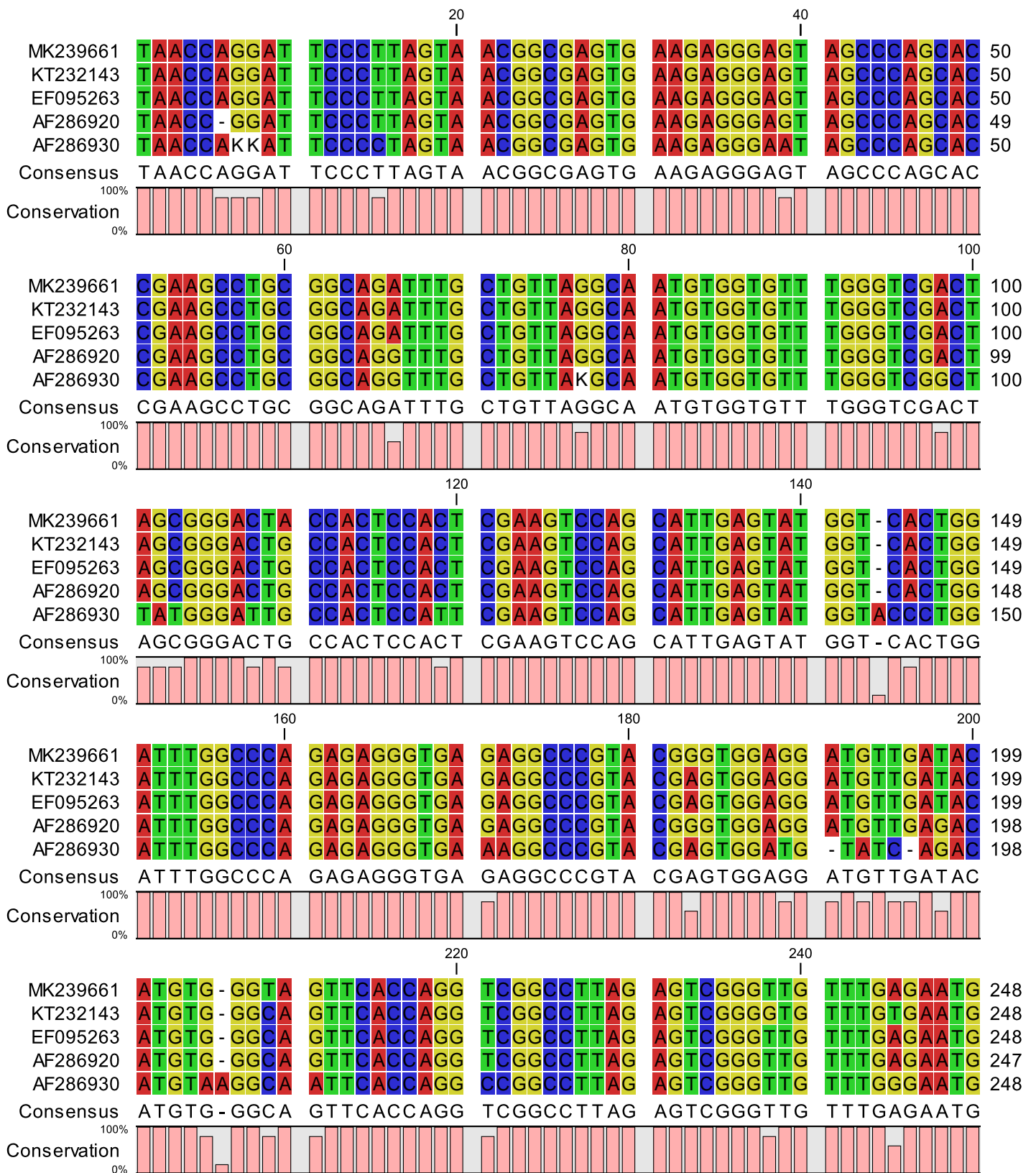

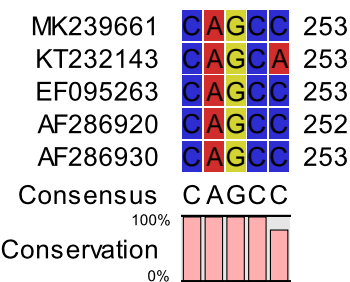

Supplement: Supplementary file 6 — Additional file 5: Figure S6. Alignment of the trimmed 28S rDNA locus (partial LSU rRNA coding sequence). [file 13071_2019_3480_MOESM6_ESM.pdf]

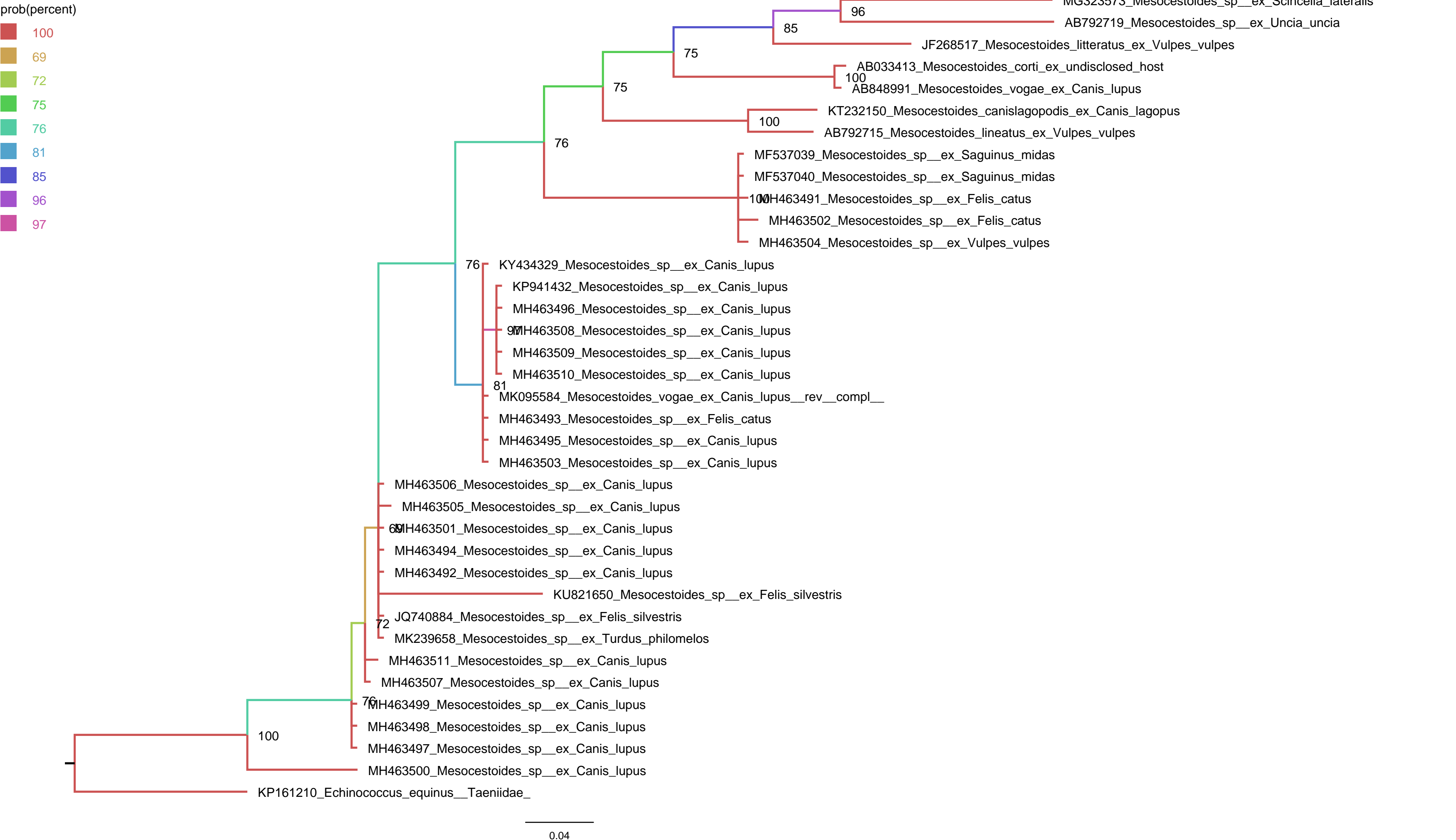

Supplement: Supplementary file 8 — Additional file 8: Figure S7. Phylogenetic tree based on Bayesian inference of the cox1 of Mesocestoides. [file 13071_2019_3480_MOESM8_ESM.pdf]

prob(percent)

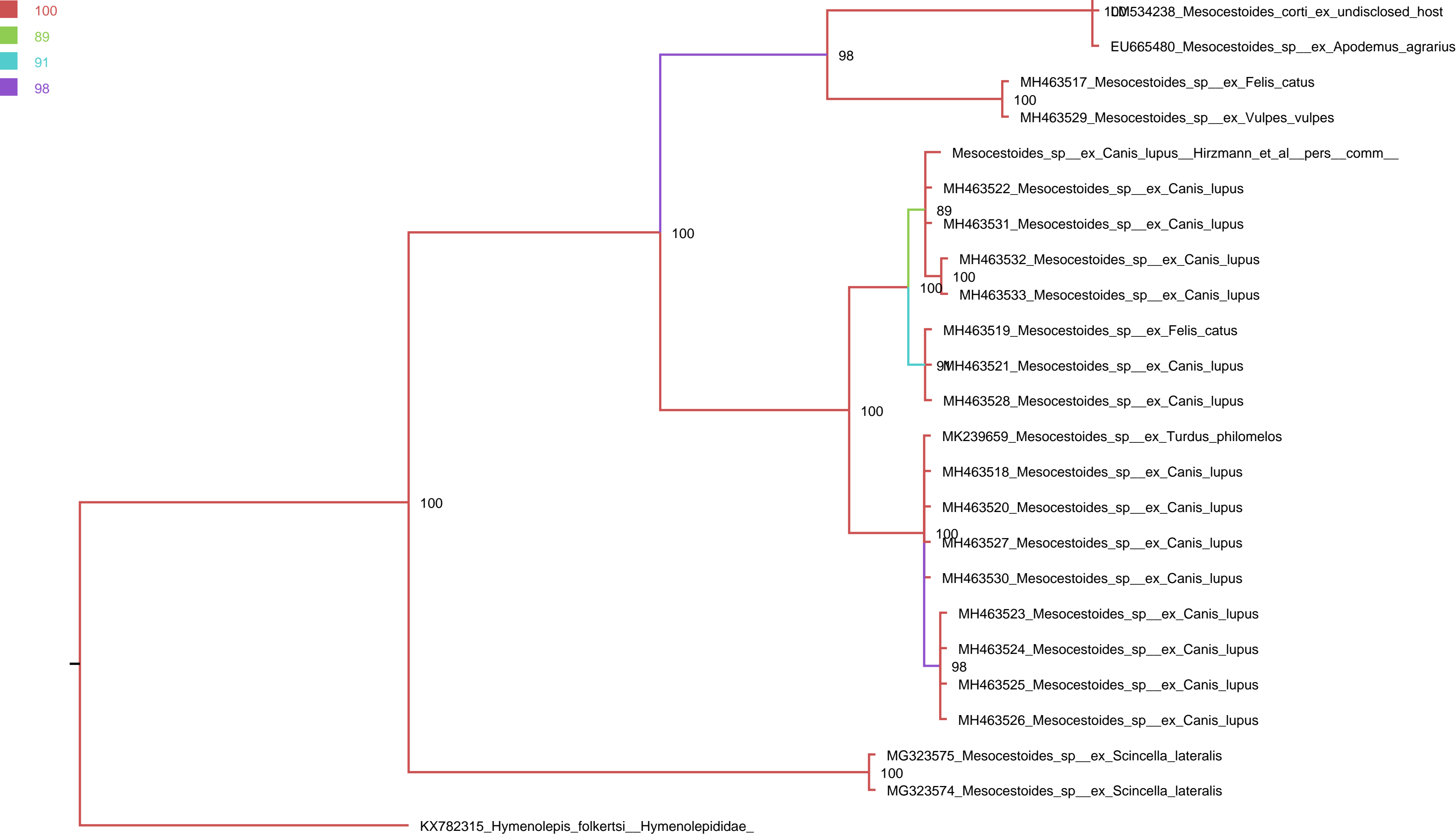

0.05

Supplement: Supplementary file 9 — Additional file 9: Figure S8. Phylogenetic tree based on Bayesian inference of the nad1 of Mesocestoides. [file 13071_2019_3480_MOESM9_ESM.pdf]

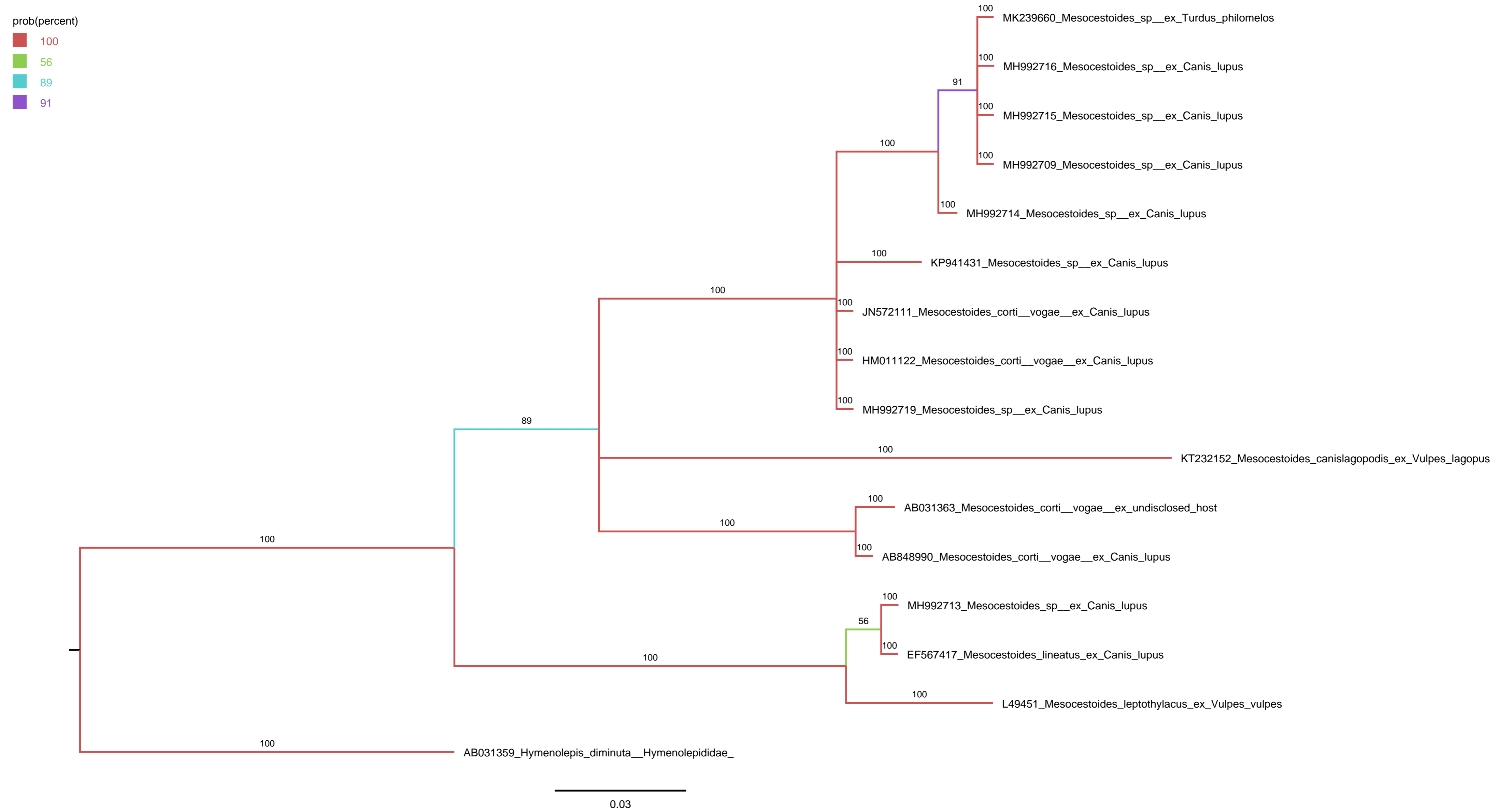

Supplement: Supplementary file 10 — Additional file 10: Figure S9. Phylogenetic tree based on Bayesian inference of the 12S rDNA of Mesocestoides. [file 13071_2019_3480_MOESM10_ESM.pdf]

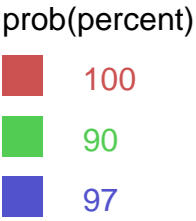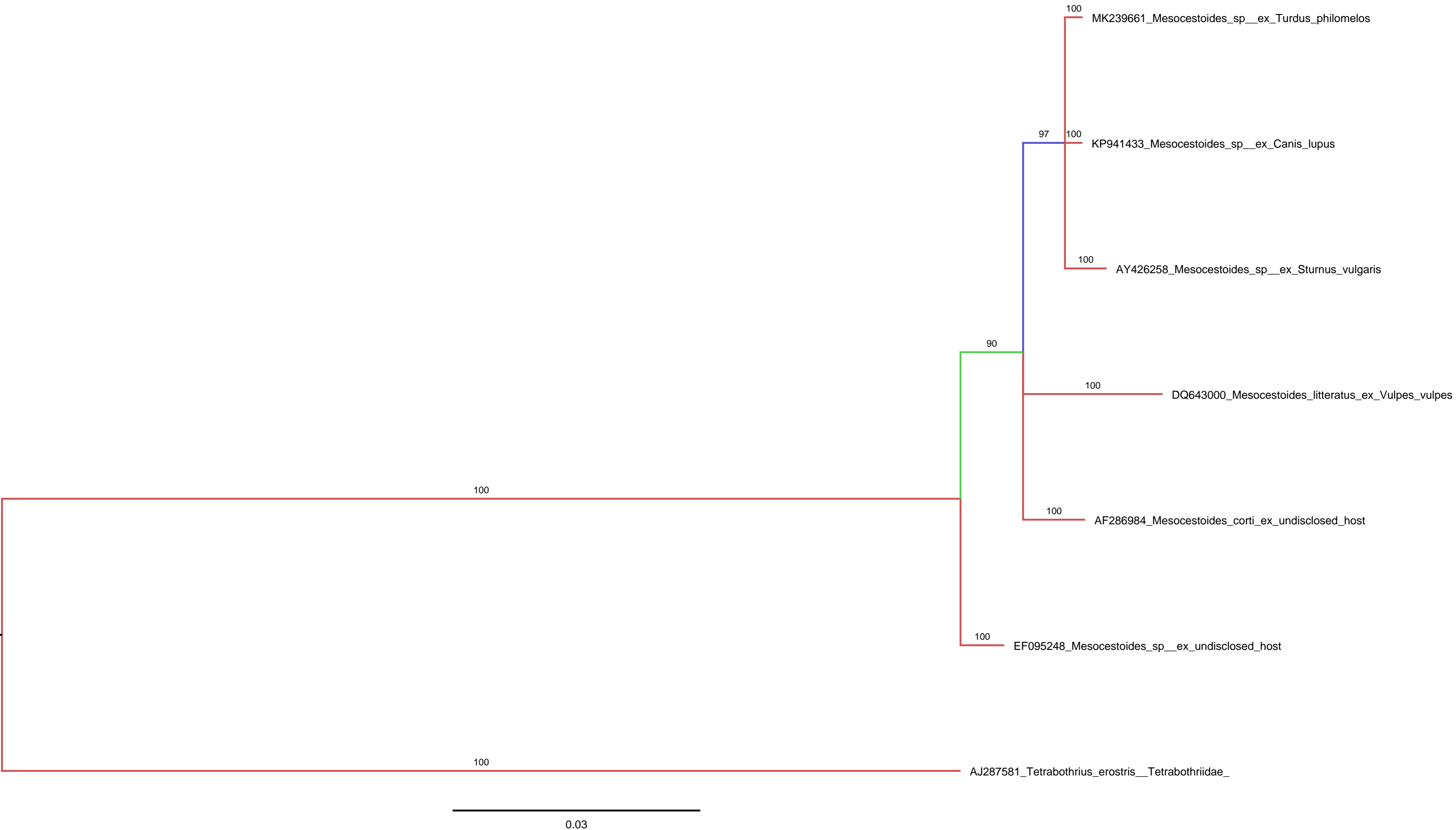

Supplement: Supplementary file 11 — Additional file 11: Figure S10. Phylogenetic tree based on Bayesian inference of the 18S rDNA of Mesocestoides. [file 13071_2019_3480_MOESM11_ESM.pdf]

prob(percent)

100

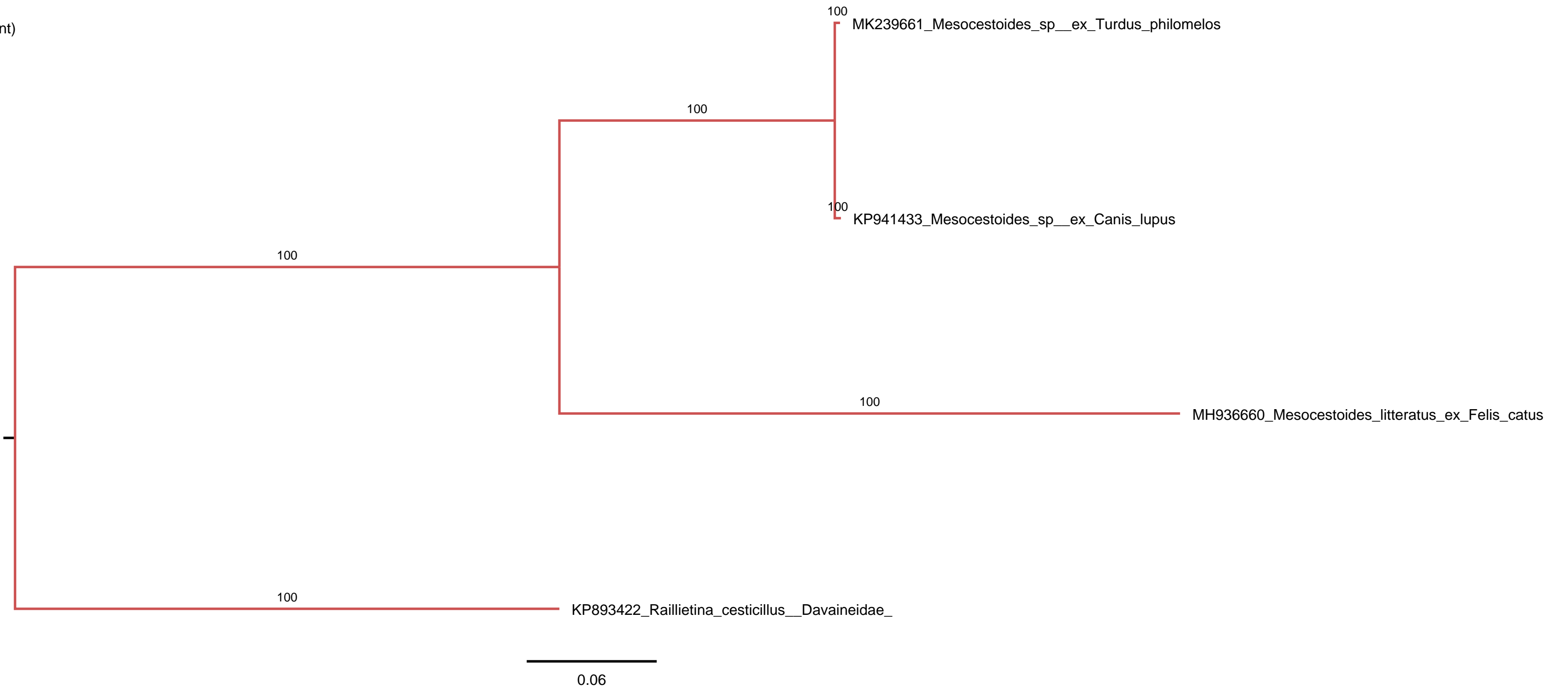

Supplement: Supplementary file 12 — Additional file 12: Figure S11. Phylogenetic tree based on Bayesian inference of the ITS1-5.8S rDNA-ITS2 of Mesocestoides. [file 13071_2019_3480_MOESM12_ESM.pdf]

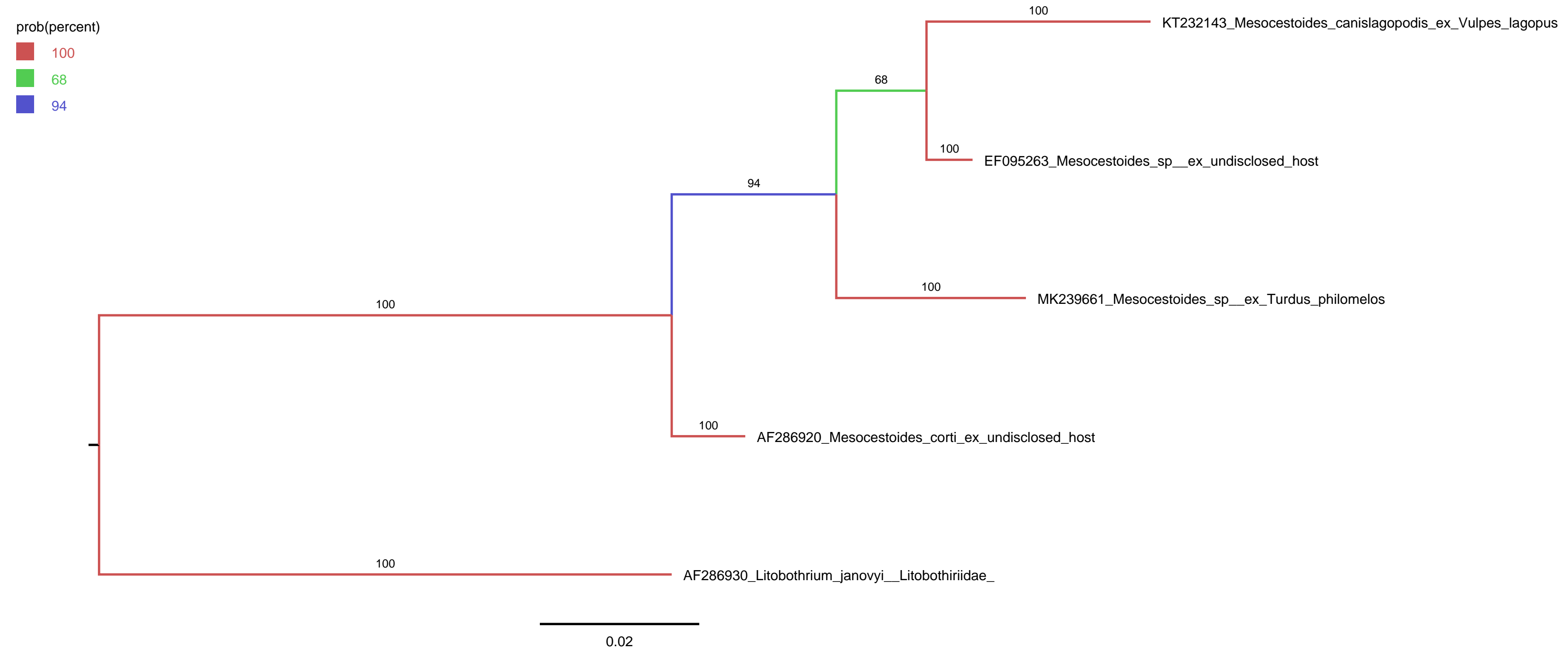

Supplement: Supplementary file 13 — Additional file 13: Figure S12. Phylogenetic tree based on Bayesian inference of the 28S rDNA of Mesocestoides. [file 13071_2019_3480_MOESM13_ESM.pdf]
