# Supplementary material for: Massive infection of a song thrush by Mesocestoides sp. (Cestoda) tetrathyridia that genetically match acephalic metacestodes causing lethal peritoneal larval cestodiasis in domesticated mammals
Source: Parasit Vectors. 2019 May 14;12:230. doi: 10.1186/s13071-019-3480-1 (PMC6518502; doi:10.1186/s13071-019-3480-1)
Supplement: Supplementary file 7 — Additional file 7: Table S1. The 12S rDNA locus sequence divergences of M. lineatus, M. canislagopodis, M. corti/vogae, M. leptothylacus and M. litteratus from the presently analyzed species. Data are shown as mean % of divergence (below the diagonal), with S.E. shown above the diagonal. [file 13071_2019_3480_MOESM7_ESM.docx]

**Additional file 7: Table S1.** The *12S* rDNA locus sequence divergences of *M. lineatus*, *M. canislagopodis*, *M. corti*/*vogae*, *M*. *leptothylacus* and *M. litteratus* from the presently analyzed species. Data are shown as mean % of divergence (below the diagonal), with S.E. shown above the diagonal.

|  | MK239660 *Mesocestoides* sp. ex *Turdus philomelos* | MH992709, 14, 15, 16 *Mesocestoides* sp. ex *Canis lupus* | MH992719 *Mesocestoides* sp. ex *Canis lupus* | *Mesocestoides* *corti* (*vogae*) | MH992731 *Mesocestoides* sp. ex *Canis lupus* | MH992713 *Mesocestoides* sp. ex *Canis lupus* | *Mesocestoides* *lineatus* | *Mesocestoides* *canislagopodis* | *Mesocestoides* *leptothylacus* | *Hymenolepis diminuta* (Hymenolepididae) |
| --- | --- | --- | --- | --- | --- | --- | --- | --- | --- | --- |
| MK239660 *Mesocestoides* sp. ex *Turdus philomelos* |  | 0.0 | 0.5 | 1.1 | 0.8 | 2.3 | 2.3 | 2.9 | 2.6 | 2.9 |
| MH992709, 14, 15, 16 *Mesocestoides* sp. ex *Canis lupus* | 0.0 |  | 0.5 | 1.1 | 0.8 | 2.3 | 2.3 | 2.9 | 2.6 | 2.9 |
| MH992719 *Mesocestoides* sp. ex *Canis lupus* | 0.7 | 0.7 |  | 0.9 | 0.7 | 2.1 | 2.1 | 2.8 | 2.4 | 2.9 |
| *Mesocestoides* *corti* (*vogae*) | 4.3 | 4.3 | 3.4 |  | 1.1 | 2.0 | 2.0 | 2.7 | 2.2 | 2.9 |
| MH992731 *Mesocestoides* sp. ex *Canis lupus* | 1.9 | 1.9 | 1.1 | 4.7 |  | 2.3 | 2.3 | 3.1 | 2.6 | 3.2 |
| MH992713 *Mesocestoides* sp. ex *Canis lupus* | 10.7 | 10.7 | 9.7 | 10.3 | 11.2 |  | 0.0 | 3.6 | 0.9 | 3.3 |
| *Mesocestoides* *lineatus* | 10.7 | 10.7 | 9.7 | 10.3 | 11.2 | 0.0 |  | 3.6 | 0.9 | 3.3 |
| *Mesocestoides* *canislagopodis* | 12.8 | 12.8 | 12.2 | 12.3 | 14.0 | 17.6 | 17.6 |  | 3.8 | 3.9 |
| *Mesocestoides* *leptothylacus* | 12.7 | 12.7 | 11.6 | 12.0 | 13.1 | 2.2 | 2.2 | 19.3 |  | 3.5 |
| *Hymenolepis diminuta* (Hymenolepididae) | 15.6 | 15.6 | 15.6 | 16.5 | 17.4 | 18.0 | 18.0 | 21.2 | 19.7 |  |
