# Supplementary material for: Massive infection of a song thrush by Mesocestoides sp. (Cestoda) tetrathyridia that genetically match acephalic metacestodes causing lethal peritoneal larval cestodiasis in domesticated mammals
Source: Parasit Vectors. 2019 May 14;12:230. doi: 10.1186/s13071-019-3480-1 (PMC6518502; doi:10.1186/s13071-019-3480-1)
Supplement: Supplementary file 14 — Additional file 14: Table S2. List of primers that were used to amplify the analyzed DNA loci. [file 13071_2019_3480_MOESM14_ESM.docx]

**Additional file 14: Table S2.** List of primers that were used to amplify the analyzed DNA loci.

| Locus | Primer name | Orientation | Primer sequence | Reference |
| --- | --- | --- | --- | --- |
| CO1 | JB3 | Forward | TTTTTTGGGCATCCTGAGGTTTAT | Bowles *et al.* (1992) |
|  | JB4.5 | Reverse | TAAAGAAAGAACATAATGAAAATG | Bowles *et al.* (1992) |
| ND1 | ND1J | Forward | AGATTCGTAAGGGGCCTAATA | Bray *et al.* (1999) |
|  | ND1J2A | Reverse | CTTCAGCCTCAGCATAATC | Morgan and Blair (1998) |
| 12S rDNA | P60-for | Forward | TTAAGATATATGTGGTACAGGATTAGATACCC | Dinkel *et al*. (1998) |
|  | P375-rev | Reverse | AACCGAGGGTGACGGGCGGTGTGTACC | Dinkel *et al*. (1998) |
| Nuclear ribosomal DNA | C for | Forward | ATGGCTCATTAAATCAGCTAT | Routtu *et al.* (2014) |
|  | A rev | Reverse | TGCTTTGAGCACTCAAATTTG | Routtu *et al.* (2014) |
|  | NSF573/19 | Forward | CGCGGTAATTCCAGCTCCA | Heneberg *et al*. (2013) |
|  | NSR1787/18 | Reverse | CGACGGGCGGTGTGTACA | Heneberg *et al*. (2013) |
|  | NSF1419/20 | Forward | ATAACAGGTCTGTGATGCCC | Heneberg *et al*. (2013) |
|  | Dig.5.8SR | Reverse | CGCAATATGCGTTCAAGATG | Heneberg *et al*. (2013) |
|  | NC13(ITS2)/F | Forward | ATCGATGAAGAACGCAGC | Heneberg *et al*. (2013) |
|  | Dig.LSU-270R | Reverse | ACAAACAACCCGACTCCAAG | Heneberg *et al*. (2013) |
|  | Dig.LSU-F1/C1 | Forward | ACCCGCTGAATTTAAGCAT | Heneberg *et al*. (2013) |
|  | 28S-1270R/22 | Reverse | CAGCTATCCTGAGGGAAACTTC | Heneberg *et al*. (2013) |
|  | Dig.LSU-F2/Rob1 | Forward | GTCCAATAGCAAACAAGTCCCG | Heneberg *et al*. (2013) |
|  | 28S-1270R/22 | Reverse | CAGCTATCCTGAGGGAAACTTC | Heneberg *et al*. (2013) |
|  | Dig.28S/894F | Forward | GTTGCGTGCGTGTCACTATT | Heneberg *et al*. (2013) |
|  | 28S-2132R/20 | Reverse | AGAGGCTGTTCACCTTGGAG | Heneberg *et al*. (2013) |
|  | 28S-1449F/27 | Forward | GGAAGTCGGAATCCGCTAAGGAGTGTG | Heneberg *et al*. (2013) |
|  | NLR2362/20 | Reverse | AGAGGCTGTTCACCTTGGAG | Heneberg *et al*. (2013) |
|  | NLF1999/19 | Forward | CCGCAKCAGGTCTCCAAG | Heneberg *et al*. (2013) |
|  | NLR2781/19 | Reverse | CCGCCCCAGYCAAACTCCC | Heneberg *et al*. (2013) |
|  | NLF2551/21 | Forward | GGGAAAGAAGACCCTGTTGAG | Heneberg *et al*. (2013) |
|  | NLR3284/21 | Reverse | TTCTGACTTAGAGGCGTTCAG | Heneberg *et al*. (2013) |

References for Table S1:

Bowles, J., Blair, D., McManus D.P. (1992). Genetic variants within the genus *Echinococcus* identified by mitochondrial DNA sequencing. *Molecular and Biochemical Parasitology*, 54, 165–173.

Bray, R.A., Littlewood, D.T.J., Herniov, E.A., Williams, B., Henderson, R.E. (1999). Digenean parasites of deep-sea teleosts: a review and case studies of intrageneric phylogenies. *Parasitology*, 119, 5125–5144.

Dinkel, A., von Nickisch-Rosenegk, M., Bilger, B., Merli, M., Lucius, R., Romig, T. (1998). Detection of *Echinococcus multilocularis* in the definitive host: coprodiagnosis by PCR as an alternative to necropsy. *Journal of Clinical Microbiology*, 36, 1871–1876.

Heneberg, P., Literák, I. (2013). Molecular phylogenetic characterization of *Collyriclum faba* with reference to its three host-specific ecotypes. *Parasitology International*, 62, 262–267.

Morgan, J.A., Blair, D. (1998). Mitochondrial ND1 gene sequences used to identify echinostome isolates from Australia and New Zealand. *International Journal for Parasitology*, 28, 493–502.

Routtu, J., Grunberg, D., Izhar, R., Dagan, Y., Guttel, Y., Ucko, M., Ben-Ami, F. (2014). Selective and universal primers for trematode barcoding in freshwater snails. *Parasitology Research*, 113, 2535–2540.
