# Supplementary material for: Massive infection of a song thrush by Mesocestoides sp. (Cestoda) tetrathyridia that genetically match acephalic metacestodes causing lethal peritoneal larval cestodiasis in domesticated mammals
Source: Parasit Vectors. 2019 May 14;12:230. doi: 10.1186/s13071-019-3480-1 (PMC6518502; doi:10.1186/s13071-019-3480-1)
Supplement: Supplementary file 15 — Additional file 15: Table S3. Summary statistics for Bayesian inference analyses performed. The data are provided for partitions with frequency ≥ 0.10 in at least one run. [file 13071_2019_3480_MOESM15_ESM.docx]

**Additional file 15: Table S3.** Summary statistics for Bayesian inference analyses performed. The data are provided for partitions with frequency ≥ 0.10 in at least one run.

| Locus | Average standard deviation of split frequencies | Maximum standard deviation of split frequencies | Average potential scale reduction factor | Maximum potential scale reduction factor |
| --- | --- | --- | --- | --- |
| CO1 | 0.0065 | 0.0211 | 1.001 | 1.013 |
| ND1 | 0.0031 | 0.0105 | 1.000 | 1.002 |
| 12S rDNA | 0.0046 | 0.0502 | 1.001 | 1.004 |
| 18S rDNA | 0.0060 | 0.0121 | 1.000 | 1.001 |
| ITS1/5.8S rDNA / ITS2 | 0.0000 | 0.0000 | 1.000 | 1.000 |
| 28S rDNA | 0.0044 | 0.0064 | 1.000 | 1.001 |
